# Target identification of small molecules using large-scale CRISPR-Cas mutagenesis scanning of essential genes

**DOI:** 10.1038/s41467-017-02349-8

**Published:** 2018-02-05

**Authors:** Jasper Edgar Neggers, Bert Kwanten, Tim Dierckx, Hiroki Noguchi, Arnout Voet, Lotte Bral, Kristien Minner, Bob Massant, Nicolas Kint, Michel Delforge, Thomas Vercruysse, Erkan Baloglu, William Senapedis, Maarten Jacquemyn, Dirk Daelemans

**Affiliations:** 1 KU Leuven Department of Microbiology and Immunology, Laboratory of Virology and Chemotherapy, Rega Institute for Medical Research, Herestraat 49, 3000 Leuven, Belgium; 2 KU Leuven Department of Microbiology and Immunology, Laboratory of Clinical and Epidemiological Virology, Rega Institute for Medical Research, Herestraat 49, 3000 Leuven, Belgium; 3 KU Leuven Department of Chemistry, Biochemistry Molecular and Structural Biology Section, Celestijnenlaan 200 G, 3001 Leuven, Belgium; 4 Department of Hematology, University Hospital Leuven, Herestraat 49, 3000 Leuven, Belgium; 5 Karyopharm Therapeutics Inc, 85 Wells Ave, Newton, MA 02459 USA

## Abstract

Unraveling the mechanism of action and molecular target of small molecules remains a major challenge in drug discovery. While many cancer drugs target genetic vulnerabilities, loss-of-function screens fail to identify essential genes in drug mechanism of action. Here, we report CRISPRres, a CRISPR-Cas-based genetic screening approach to rapidly derive and identify drug resistance mutations in essential genes. It exploits the local genetic variation created by CRISPR-Cas-induced non-homologous end-joining (NHEJ) repair to generate a wide variety of functional in-frame mutations. Using large sgRNA tiling libraries and known drug–target pairs, we validate it as a target identification approach. We apply CRISPRres to the anticancer agent KPT-9274 and identify nicotinamide phosphoribosyltransferase (NAMPT) as its main target. These results present a powerful and simple genetic approach to create many protein variants that, in combination with positive selection, can be applied to reveal the cellular target of small-molecule inhibitors.

## Abstract (web summary)

Cancer therapy drugs are designed to target genetic vulnerabilities, but loss-of-function screens often fail to identify essential genes in drug mechanism studies. Here the authors demonstrate CRISPRres, which exploits in-frame variation generated by indel formation to discover gene-drug interactions.

## Introduction

Identifying the cellular target of a chemical hit with valuable bioactivity is a crucial step in drug discovery and development^1^. However, unraveling the molecular target of a small-molecule drug still remains a challenging, laborious, and complex process. Although many target deconvolution methods^2,3^, such as chemical proteomics, have successfully been applied, they often reveal more than one plausible candidate target protein and carry the risk of identifying interactions that are not related to the compound’s activity. The gold standard proof for a drug’s target is the identification of functional mutations that confer resistance in a cellular context. For this reason, genetic screens in particular, are very powerful tools for drug mechanism of action studies^4^. However, current screens either are not well suited to identify essential genes or require whole-exome sequencing combined with complex bio-informatics to deconvolute the relevant drug resistance conferring mutations. For example, loss-of-function approaches have been applied to obtain drug resistance^5–8^, but innately lack the ability to comprehensively detect gain-of-function mutations and fail to nominate essential proteins involved in drug mechanism of action. Classical step-wise drug resistance selection enables selection of gain-of-function mutations but is laborious^9^ and often results in off-target multi-drug resistance^10^. Recently, chemical mutagenesis to increase the occurrence of single-nucleotide variants has been described^11^. However, until now, this chemical mutagenesis approach has only been applied to identify loss-of-function resistance mutations to the prototype acute myeloid leukemia drug 6-thioguanine. It remains to be investigated whether this approach can also detect gain-of-function resistance mutations. Another bottleneck of these general random mutagenesis approaches is the discovery of the resistance mutations. They require sequencing of the large human exome in individual clones^11–14^ while the genomic heterogeneity of the cell line makes the deconvolution of the relevant resistance-conferring mutations especially challenging. As such, the field would greatly benefit from an approach that can accelerate the drug resistance selection process and simplify subsequent identification of the relevant drug resistance mutations. Moreover, because many cancer drugs target essential proteins, there is a strong need for a method that can easily generate and identify drug resistance mutations in essential genes.

Drawing a parallel to the use of UV-mediated double-strand breaks (DSBs) to enhance mutagenesis^15^, we reasoned that introduction of DSBs by targeted endonucleases, such as SpCas9, and the subsequent error-prone repair via non-homologous end-joining (NHEJ) may be exploited for rational protein mutagenesis to facilitate drug resistance selection. Here, we describe a CRISPR-based method, entitled CRISPR-induced resistance in essential genes (CRISPRres), to rapidly acquire and identify functional drug resistance mutations. We show that large-scale CRISPR single-guide RNA (sgRNA) gene tiling libraries can be applied as a genetic screening approach in cancer cells to identify the molecular target of a chemical inhibitor. Finally, we also demonstrate that the methodology is compatible with the class 2 type V AsCpf1 CRISPR system, increasing the resolution of the method.

## Results

### Rapid generation of drug-resistant variants with CRISPR-Cas9

**Fig. 1:**
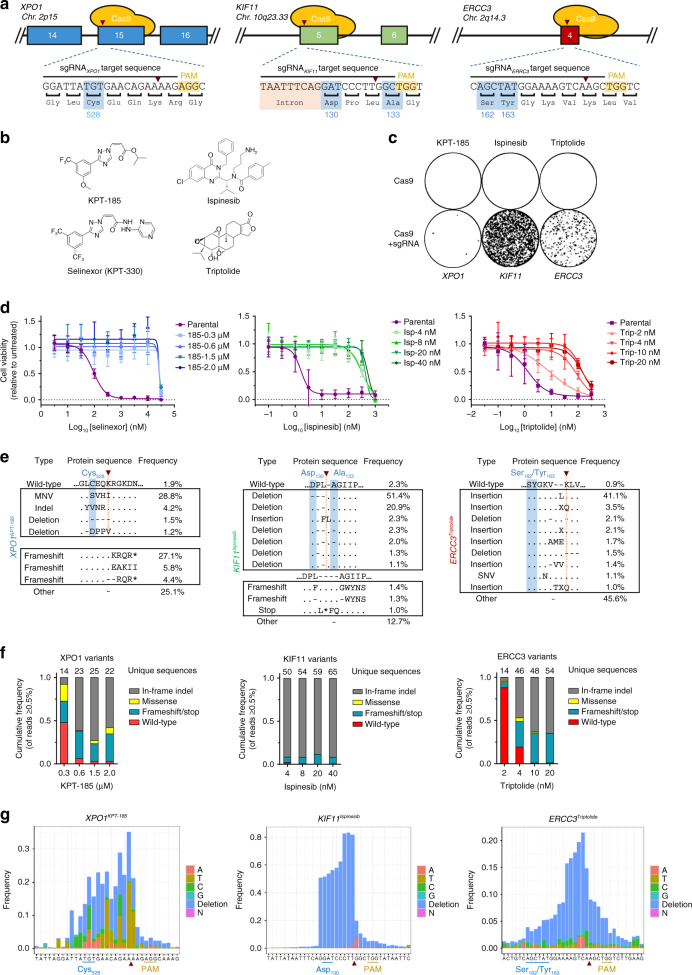
Spontaneous genetic variation generated by CRISPR-Cas9-induced NHEJ repair facilitates rapid selection of drug resistant protein variants. **a** Representation of sgRNAs targeting resistance hotspots for KPT-185/selinexor (*XPO1* codon C528), ispinesib (*KIF11* codons D130 and A133), and triptolide (*ERCC3* codons S162 and Y163). The SpCas9 cleavage site is indicated (red arrowhead) and residues conferring drug resistance when altered are hightlighted in blue. **b** Chemical structures of the antineoplastic agents. **c** SpCas9-induced NHEJ repair at resistance hotspots facilitates rapid selection of resistant colonies. Cells were transfected with SpCas9 plasmid (top) or co-transfected with SpCas9 and sgRNA expressing plasmids (bottom). KPT-185 (300 nM), ispinesib (4 nM), or triptolide (10 nM) were added 48 h after transfection and selection was maintained for 7–10 days before visualization. The experiment was performed with four different compound concentrations (KPT-185: 0.3, 0.6, 1.5, 2 μM; ispinesib: 4, 8, 20, 40 nM; triptolide: 2, 4, 10, 20 nM) and replicated at least once (see Supplementary Figs. 1, 2, and 3), but only one representative concentration is shown. **d** Cell viability assays of wild-type (parental) and the different resistant cell populations obtained after selection of mutagenized cells with four different concentrations of compound. Data points are normalized relative to untreated cells and represent mean ± s.d. obtained from three experiments performed in triplicate. **e** Amino-acid sequence variants, determined by targeted amplicon sequencing analysis with CrispRVariants present in the resistant cell pools described in **c**. The wild-type sequence is shown for reference and resistance hotspot residues are highlighted in blue. The SpCas9 cleavage site is indicated (red arrowhead). For a complete list of amino-acid variants from all resistant cell pools and replicates see Supplementary Figs. 1a, 2, 3. **f** The relative abundance of all alleles with a read frequency ≥0.5% is shown and categorized per sample into four different mutation types. The values represent averages of two (*XPO1*, *ERCC3*) or three (*KIF11*) experiments for each concentration of the compound. **g** Single-nucleotide variant occurrence at the sgRNA target sites observed across all mutagenized and selected cell pools from **c**. The red arrowhead indicates the Cas9 cut site. The values represent averages of eight (*XPO1*, *ERCC3*) or 12 (*KIF11*) experiments

To develop the method, we first designed sgRNAs targeting known resistance hotspots in genes sensitive to three cancer drugs: KPT-185, a preclinical analog of the XPO1 inhibitor selinexor^16–19^, ispinesib, an antineoplastic kinesin-5 (KIF11) inhibitor^13,20^, and triptolide, an antiproliferative agent targeting ERCC3^14,21^ (Fig. 1a, b). The respective sgRNAs were transiently expressed together with SpCas9 in chronic myeloid leukemia-derived HAP1 cells which were then treated with four different concentrations of the corresponding drug. Within a few days of treatment, colonies that were resistant to the drugs appeared on the culture plates (Fig. 1c, d). Next-generation sequencing of the targeted hotspot loci of these resistant colonies revealed known as well as many novel resistant protein variants (Fig. 1e and Supplementary Figs. 1a, 2, and 3). Mutations were mainly localized within 17 bp upstream of the SpCas9 cleavage site on the non-target strand and consisted of insertions, deletions, and missense mutations (Fig. 1f, g). The majority of the sequences consisted of in-frame mutations, but some frameshift and nonsense mutations were also detected. Because the targeted genes are essential for survival, this suggests that some of the cells had turned diploid during the experiment, a phenomenon known to occur spontaneously in HAP1 cells^22^. For XPO1, more than 40 different in-frame variants containing a mutation or deletion of the C528 residue were detected (Fig. 1e left panel and Supplementary Fig. 1a). This is in agreement with the fact that this single cysteine residue is absolutely essential for KPT-185/selinexor activity^16–18^. However, until now, only a single serine substitution had been reported to confer cancer cells with resistance to these drugs^16^. As we were surprised to see that an essential protein such as XPO1 can accommodate such a wide variety of diverse mutations in a functional domain, we derived different single-cell clones from the original compound selected cell pools to confirm the resistance-conferring mutations by Sanger sequencing and cell viability in the presence of the drug (Supplementary Fig. 1b, c). For KIF11, the majority of the sequencing reads contained a 9 bp deletion from codon D130 to L132 (Fig. 1e middle panel and Supplementary Fig. 2). Interestingly, the majority of sequences shared a deletion or mutation of L132, which appears to be sufficient for drug resistance per se. Mutation of residue A133, known to confer ispinesib resistance^13^, was not readily detected, which might be explained by the fact that the codon for A133 is at the 3′ end of the SpCas9 cut site. Indeed, mutations were mainly observed upstream of the SpCas9 cut site, away from the NGG protospacer adjacent motif (PAM) (Fig. 1g middle panel). Interestingly, in contrast to the selinexor resistance mutations, almost all ispinesib-resistant sequence alterations were deletions. For ERCC3, we mainly identified in-frame insertions and some deletions between codons K165 and K167 (Fig. 1e right panel and Supplementary Fig. 3). We also targeted an additional resistance hotspot at *ERCC3* codon D54, which resulted in in-frame deletions of 1–8 amino acids consistent with previous observations^14^ (Supplementary Fig. 4). To confirm that drug resistance was obtained by targeted CRISPR-Cas9-mediated mutagenesis and not by multi-drug resistance, the drug-resistant colonies were treated with all three drugs and no cross-resistance between the three different drug-resistant cell pools was observed (Supplementary Fig. 5). To demonstrate that this mutagenesis methodology can be broadly applied to other cell types, similar results were obtained in the pseudodiploid acute pro-myelocytic leukemia HL-60 and colon cancer HCT 116 cell lines (Supplementary Figs 6 and 7).

To validate the observed drug resistance mutations, we reinstalled one protein variant for each drug (XPO1^C528S,E529V,Q530H,K531I^; KIF11^L132Δ^; ERCC3^V166_K167insL^) into its native locus in parental cells using CRISPR-Cas9-mediated homology-directed repair (HDR) of a ssDNA donor template (Supplementary Fig. 8a). Silent mutations not identified in our original selection were included to control for HDR (Supplementary Fig. 8b). All three donor templates provided strong protection against respective drug treatment (Supplementary Fig. 8a, c), validating the results obtained by the targeted CRISPR-Cas9-induced NHEJ-based mutagenesis. We also assessed protein function of the resistant variants and how this is affected by the respective drugs. We first visualized the XPO1-dependent nuclear export of a GFP protein fused to the nuclear localization signal (NLS) of SV40 and the nuclear export signal (NES) from the prototype XPO1 cargo protein kinase inhibitor (PKI). This fusion protein actively shuttles between the nucleus and cytoplasm but localizes in a XPO1-dependent manner to the cytoplasm in steady state (Supplementary Fig. 8d panel a)^18^. CRISPR-Cas9-mediated knockout of XPO1 in both wild-type and mutant cells resulted in nuclear accumulation of the reporter protein, demonstrating functionality of the resistant XPO1 protein variant (Supplementary Fig. 8d panel b). Upon inhibition of the XPO1-mediated nuclear export by selinexor, the GFP fusion protein accumulated in the nucleus of wild-type cells, while in XPO1^C528S,E529V,Q530H,K531I^ mutant cells its localization remained unaffected (Supplementary Fig. 8d panel c), again demonstrating that the mutation confers resistance to inhibition of XPO1 function by selinexor. As an additional confirmation of drug resistance, we further demonstrated that the resistance mutations prevent the drug from binding directly to XPO1 by the absence of pull-down of mutant XPO1 protein with a biotinylated analog of selinexor (Supplementary Fig. 8e). For the KIF11^L132Δ^ resistance mutation, the formation of mitotic spindles in genome-edited KIF11^L132Δ^ cells was similar to that in the wild-type cells (Supplementary Fig. 8f panel a). Knockout of KIF11 disrupted bipolar spindle formation in both wild-type and KIF11^L132Δ^ mutant cells (Supplementary Fig. 8f panel b), demonstrating that the mutant protein is functional. Furthermore, the KIF11^L132Δ^ mutation protected cells from monopolar mitotic spindle formation induced by ispinesib treatment (Supplementary Fig. 8f panel c).

Taken together, these results demonstrate that the spontaneous genetic variation generated during NHEJ repair at the locus of CRISPR-SpCas9-mediated DSBs can be exploited to significantly accelerate the selection of functional drug resistance mutations; a finding independently confirmed by Ipsaro et al.^23^ and Donovan et al.^24^.

### Target identification by sgRNA tiling of multiple genes

To further investigate whether this approach can be applied as a genetic screen to directly identify the molecular target of a chemical inhibitor, we designed a lentiviral tiling sgRNA library spanning all possible NGG PAM sites in the coding sequence from nine different genes (2,209 sgRNAs), including kinesin-5 (*KIF11*) (Supplementary Data 1). HAP1 cells stably expressing SpCas9 were transduced with this lentiviral sgRNA library and subsequently treated with ispinesib for a period of 14 days (Supplementary Fig. 9a). Drug-resistant colonies formed rapidly and were resistant to ispinesib (Supplementary Fig. 9b). Four sgRNAs, which all targeted *KIF11*, were highly enriched in the pool of resistant clones (Supplementary Fig. 9c and d, and Supplementary Data 2). When transfected separately, these sgRNAs were able to confer HAP1 cells with ispinesib resistance, with the sgRNAs targeting codon A133 being the most efficient (Supplementary Fig. 9e). The underlying resistance mutations at the genomic locus targeted by this sgRNA consisted of multiple in-frame variations around the *KIF11* A133 codon (Supplementary Fig. 9f), consistent with previous results (Fig. 1e middle panel), and map to the ispinesib-binding site. These results show that CRISPR-Cas9 mutagenesis scanning on a pool of genes can be used to fish out both the target protein and the molecular interaction site of a small-molecule inhibitor.

**Fig. 2:**
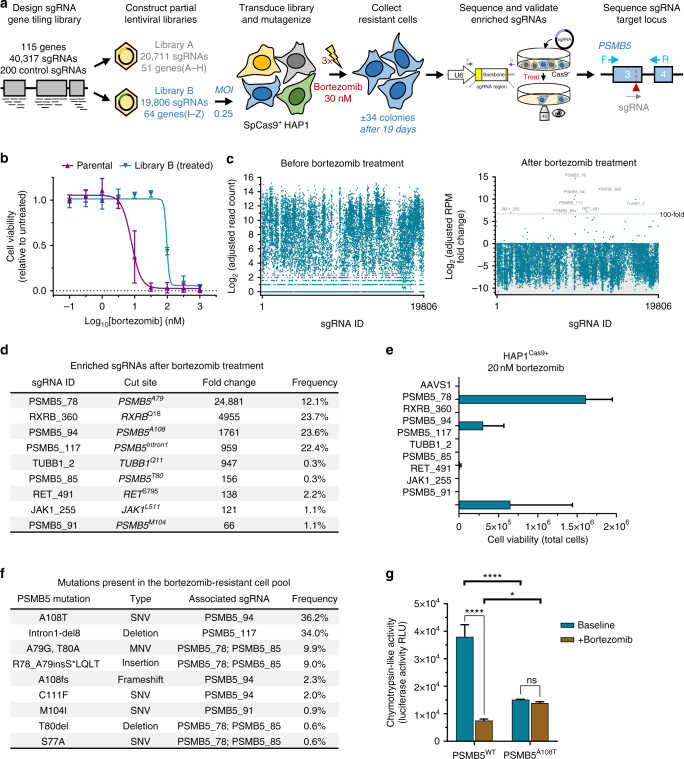
Validation of CRISPR-Cas-mediated mutagenesis scanning as a target identification approach using large sgRNA tiling libraries. **a** Overview of the workflow for the CRISPR-Cas9-based target identification screen used for bortezomib. Two lentiviral sgRNA sublibraries, together tiling 115 genes targeted by FDA-approved anticancer drugs, were constructed (A: *ABL1* through *HDAC9*; B: *IFNAR1* through *VEGFB*). Sublibrary B was transduced into HAP1 cells stably expressing SpCas9, enriched with puromycin and treated with 30 nM bortezomib for 19 days. sgRNAs in the surviving cells were then sequenced and validated. **b** Cell viability of parental and the pool of bortezomib-resistant HAP1 cells in the presence of different concentrations of bortezomib. Data points represent mean ± s.d. obtained from three experiments performed in triplicate. **c** Representation of the different sgRNAs in cells before (after puromycin selection) and after treatment with 30 nM bortezomib as determined by EdgeR analysis of next-generation sequencing data. Each dot represents a different sgRNA. A value of 1 was added to each read count to facilitate log transformation. **d** Overview of the sgRNA hits enriched in the bortezomib-surviving cells. **e** Validation of the individual sgRNA hits by assessing their ability to induce bortezomib resistance. Individual sgRNAs were cloned into an expression vector and transfected separately into SpCas9^+^ HAP1 cells. Cells were treated with 20 nM bortezomib for 8 days and then counted using trypan blue exclusion. An sgRNA targeting the *AAVS1* safe harbor locus was used as negative control. The columns represent mean ± s.d. obtained from two separate experiments. **f** PSMB5 amino-acid variants present in the pool of bortezomib-resistant cells as determined by CrispRVariants analysis of next-generation targeted amplicon sequencing. **g** PSMB5 chymotrypsin-like activity as measured in parental HAP1 and a single-cell-derived PSMB5^A108T^ mutant obtained from the pool of bortezomib-resistant cells. Cells were untreated or treated for 2 h with 12.5 nM bortezomib. Values represent mean ± s.d. obtained from a single experiment performed in triplicate; *p-value < 0.05 *, ****p-value < 0.0001 (two-way ANOVA with Bonferroni correction for multiple testing), ns not significant, RLU relative light unit

To extend the use of this approach to drugs for which resistance has not yet been developed or for which the target is not known, we designed two complementary lentiviral sgRNA tiling libraries containing the target genes of FDA approved (115 genes, divided in two subpools A and B of ±20,000 sgRNAs each) or investigational antineoplastic drugs (75 genes, divided in two subpools C and D containing ±12,000 sgRNAs each) (Supplementary Data 3 and 4). As validation of these libraries, we applied sublibrary B to SpCas9^+^ HAP1 cells to obtain resistance against the proteasome inhibitor bortezomib, which targets proteasome subunit beta type-5 (PSMB5) (Fig. 2a). Surviving colonies were harvested within 19 days after treatment. These were resistant to bortezomib treatment (Fig. 2b) and documentation of the sgRNAs present in the resistant cell population revealed four sgRNAs targeting known *PSMB5* resistance hotspots amongst other sgRNAs (Fig. 2c, d, Supplementary Data 5). Validation of the >100-fold enriched sgRNAs revealed that *PSMB5* was the sole gene that, when mutagenized, conferred bortezomib resistance (Fig. 2e, f). Deep sequencing of the genomic region targeted by these sgRNAs identified multiple in-frame mutations in *PSMB5* (Fig. 2f). Most of these mutations are similar to known drug resistance mutations and map to the bortezomib binding site^25,26^. More specifically, the most enriched mutation (A108T) alters a residue known to bind bortezomib directly. Other well-known bortezomib drug resistance mutations (C111F and M104I) were identified, as well as new and earlier described mutations around threonine 80 (S77A, A79G, T80A, T80del), which is also implicated in the binding of bortezomib. One peculiar intronic mutation (deletion of the nucleotides 5′-GCCCCCTT) was identified 11 bp downstream of the exon1–intron1 boundary. Although we could not validate the associated sgRNA (PSMB5_117) (Fig. 2e), another recent study using targeted mutagenesis of *PSMB5* also identified an intronic point mutation in the first intron of *PSMB5* in bortezomib-resistant cells^25^. To further investigate the effect of bortezomib on mutated cells, we obtained a single-cell-derived A108T mutant from the pool of resistant cells and examined chymotrypsin-like proteasome activity in these cells (Fig. 2g). Baseline proteasome activity in mutant cells was decreased when compared to the wild-type (*p* < 0.0001) but treatment with bortezomib did not inhibit proteasome activity, while it was severely impaired in the parental wild-type (*p* < 0.0001).

These results clearly validate this tiling library and demonstrate the feasibility of a SpCas9-directed mutagenesis scanning strategy for target deconvolution on a larger scale.

### Identification of the cellular target of KPT-9274

**Fig. 3:**
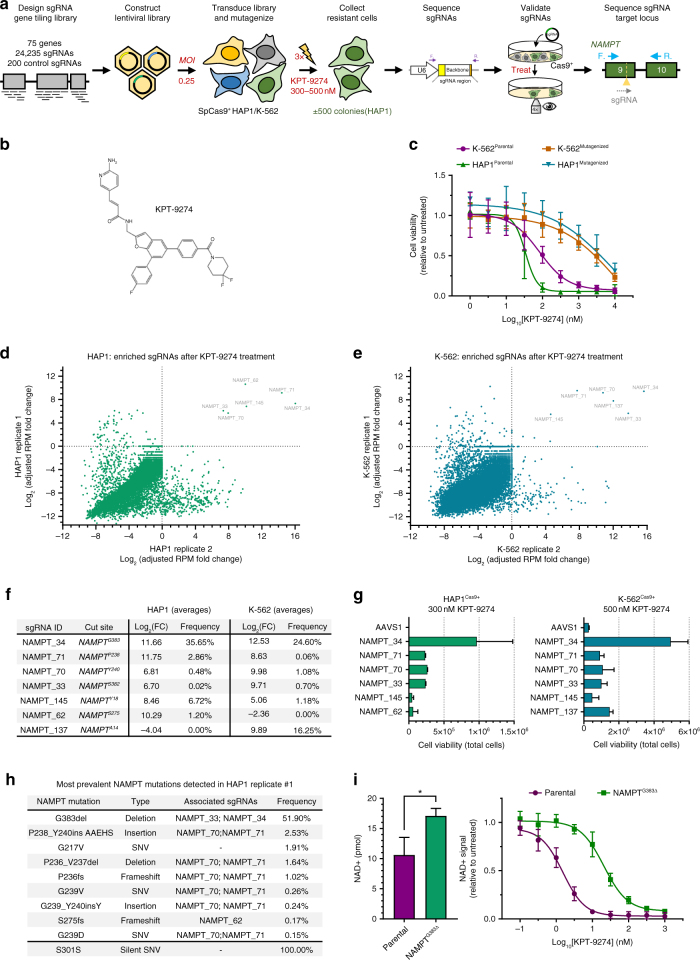
Identification of NAMPT as the cellular target of KPT-9274 using a CRISPR-Cas9 tiling library. **a** Experimental workflow for the CRISPR-Cas9-based target identification screen for KPT-9274. HAP1 or K-562 SpCas9^+^ cells were transduced with the investigational target gene library spanning 75 genes, enriched by puromycin selection and treated with KPT-9274. Surviving cells were then harvested and the sgRNAs were sequenced. The experiment was performed in duplicate for both cell lines. **b** Chemical structure of KPT-9274. **c** Resistance profile of parental cells and transduced cell populations that survived treatment with KPT-9274. Cell viability was measured in the presence of increasing concentrations of KPT-9274 and was adjusted to the untreated control. Data points represent mean ± s.d. obtained from two (HAP1) or five (K-562) experiments performed in triplicate. **d** Enrichment scores of the sgRNAs present in transduced HAP1 cells after treatment with KPT-9274 (300 nM). Fold change was determined for each sgRNA by taking the log_2_ of the reads per million (RPM) after treatment added by 1 (adjusted) divided by the adjusted RPM before treatment. Read counts were determined using EdgeR and each dot represents an sgRNA. **e** Enrichment scores of the sgRNAs present in transduced K-562 cells after treatment with KPT-9274. **f** Overview of the enriched sgRNAs present in the transduced cells after treatment with KPT-9274. Values represent averages of the replicate experiments for each cell line. FC: adjusted fold change. **g** sgRNAs identified in the screen were validated by assessing their ability to induce drug resistance. Each sgRNA was transfected separately and cells were treated with KPT-9274 for four (HAP1) or six (K-562) days. Surviving cells were counted using trypan blue exclusion. A safe-targeting sgRNA (AAVS1) was included as negative control. The columns indicate mean ± s.d. obtained from two experiments. **h** Overview of NAMPT mutations detected in KPT-9274-resistant cells from HAP1 replicate #1 (see also Supplementary Data 7). **i** NAD^+^ levels in parental and HDR-edited NAMPT^G383del^ HAP1 cells treated with increasing concentrations of KPT-9274. The left panel shows the untreated baseline NAD^+^ levels. Two experiments were performed in duplicate and values represent mean ± s.d. *p-value < 0.05 two-tailed Mann–Whitney *U* test

Next, we applied the approach to the clinical stage anticancer compound KPT-9274, an orally bioavailable small molecule with potent activity against different cancer types. Based on chemical proteomics experiments, KPT-9274 has been suggested to interact with the p21-associated kinase 4 (PAK4)^27^ and inhibition of NAD^+^ biosynthesis has also been reported^28^. However, the causal association between these activities and cancer cell sensitivity to KPT-9274 remains to be investigated. Therefore, we applied our sgRNA tiling library containing 75 target genes of investigational antineoplastic drugs to HAP1 and chronic myelogenous leukemia K-562 cells stably expressing SpCas9 (Fig. 3a and Supplementary Data 4). Cells were pulsed three times with KPT-9274 (Fig. 3b) and colonies that were resistant to KPT-9274 (Fig. 3c) appeared within 7–14 days post treatment. In both cell lines, six sgRNAs, all targeting nicotinamide phosphoribosyl transferase (*NAMPT*), were enriched in the resistant cell pools (Fig. 3d, e, and Supplementary Data 6). From these, five were identical in both cell lines (Fig. 3f). One sgRNA, targeting *NAMPT* codon G383, was by far the most enriched in both HAP1- and K-562-resistant cell pools and represented between 22% and 38% of total sgRNAs. In addition, an sgRNA targeting *NAMPT* that was present as little as 0.02% could also be detected. All enriched sgRNAs could be confirmed to confer resistance when transfected separately (Fig. 3g). The same KPT-9274 resistance could also be elicited in pancreas carcinoma MIA PaCa2 and multiple myeloma OPM-2 cells by co-transfection of the *NAMPT* sgRNAs with SpCas9 (Supplementary Fig. 10a). The original KPT-9274-resistant cell pools were also cross-resistant to the NAMPT inhibitor FK866^29^ (Supplementary Fig. 10b, c), and the same NAMPT sgRNAs identified in the screen were able to confer HAP1 cells with resistance to FK866 (Supplementary Fig. 10d). Moreover, addition of β-nicotinamide mononucleotide, the product of the enzymatic reaction catalyzed by NAMPT, to parental wild-type cells provided similar protection against KPT-9274-induced cytotoxicity as the *NAMPT* sgRNAs from the screen (compare Fig. 3c with Supplementary Fig. 10e), further pinpointing NAMPT as a key cellular target of KPT-9274.

Next, we identified the underlying resistance mutations present in the mutagenized population of cells obtained from the sgRNA scanning screen. As some of the enriched sgRNAs had overlapping target sequences (NAMPT_34 and NAMPT_33; NAMPT_137 and NAMPT_145), only four domains within the NAMPT protein were effectively targeted. Deep sequencing of *NAMPT* at these four domains revealed a variety of mutations (Fig. 3h and Supplementary Data 7). The G383del mutation was by far the most prevalent, ranging from 10 to 52%, and was the only mutation identified throughout all samples. The P236_V237del mutation was present only in the two replicates from the HAP1 experiment, while the G381_G384del mutation was only present in the K-562 replicates. Sequencing of the first *NAMPT* exon, which is targeted by the NAMPT_145 and NAMPT_146 sgRNAs, proved to be difficult due to the high GC content in the flanking introns. However, in some samples we did manage to identify I11_K19del, S17del, and Y18del mutations, although we could not quantify these mutations. Other in-frame mutations, including P238_Y240insAAEHS, S275T, S382_G385del, and some frameshift mutations, were identified in individual replicates. Frameshift mutations are most probably present in hemizygous cells since knockout of NAMPT is lethal in HAP1 and K-562 cells (Supplementary Fig. 11d). We also identified some low prevalent NAMPT mutations that did not localize to the target sites of the enriched sgRNAs. Three of these mutations (G217E, G217V, G217R) alter the G217 residue that has been identified earlier to confer resistance to NAMPT inhibitors^30^. These mutations were found in one of the two replicates and were likely already present in the heterogenous parental population or arised during the experiment. Finally, one known SNP (S301S; rs2302559 (dbSNP)) was also detected in both replicate HAP1 screens.

We further validated the G383del mutation, which was the only mutation detected in both HAP1 and K-562 replicates, by reinstalling it into its native locus by CRISPR-induced HDR of parental HAP1 cells (Supplementary Fig. 11a). HDR-edited cells were resistant to KPT-9274 treatment and were also cross-resistant to FK866 (Supplementary Fig. 11b, c), again providing further evidence for NAMPT as the key cellular target of KPT-9274. To examine the function of NAMPT protein containing the G383del mutation, cellular NAD^+^ levels were measured in absence and presence of KPT-9274. Baseline NAD^+^ levels were slightly elevated in mutant cells (Fig. 3i left panel), and treatment of parental cells with KPT-9274 decreased NAD^+^ levels rapidly, while the G383del mutation conferred resistance (Fig. 3i right panel).

**Fig. 4:**
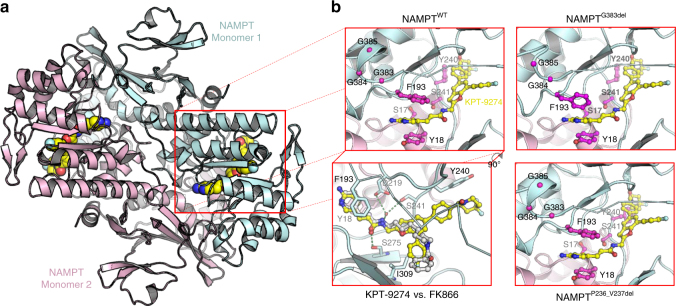
Co-crystal structure of NAMPT dimer with KPT-9274. **a** The crystal structure of NAMPT dimer with KPT-9274 is depicted as a cartoon with the NAMPT monomers shown in light blue and pink and KPT-9274 as yellow spheres. **b** A sideways blow-up (top left) shows the drug-binding site with KPT-9274 depicted in yellow carbons. A second blow-up (down left) shows the overlap between KPT-9274 and FK866. The pi stacking of KPT-9274 between the Y18 and F193 matches the binding mode of FK866 and important hydrogen bonds, depicted as green dashed lines (between S275 and protein-bound water coordinated by D219 and S241), overlap between the two compounds. The right panels show the influence of the G383del (top) and P236_V237del (bottom) resistance mutations

Next, to unambiguously validate our results and corroborate the validity of the conclusions taken from the mutagenesis scanning approach, we crystallized a NAMPT dimer in complex with KPT-9274 (Fig. 4a and Supplementary Fig. 12) (PDB: 5NSD) and explained the identified resistance mutations by modeling (Fig. 4b). Crystallographic analysis of the binding mode revealed that two KPT-9274 molecules are bound to the NAMPT dimer (Fig. 4a). The binding mode is largely similar to that of FK866 (Fig. 4b left lower panel)^29^. In more detail, the amino-pyridinyl of KPT-9274 is coordinated via pi-stacking interactions between tyrosine 18 and phenylalanine 193. Furthermore, the amide linker is well coordinated via hydrogen-bonding interactions and the free electron pairs of the amide oxygen are stabilized by a hydrogen bond originating from serine 275. The nitrogen forms a hydrogen bond with a water molecule that further interacts with the carboxyl group of aspartate 219 and the hydroxyl group of serine 241. At the solvent exposed regions of the binding site, the chemical composition of KPT-9274 and the interactions with the NAMPT protein are different from FK866. The KPT-9274 core consists of a rigid benzofuran moiety, contrary to the flexible carbon chain linker in FK866 (compare Fig. 3b and Supplementary Fig. 10b). KPT-9274 is branched and binds to two different solvent exposed patches whereas FK866 only occupies a single hydrophobic site. The first hydrophobic patch outlined by the hydrophobic sidechains of isoleucine 309, isoleucine 351, and alanine 379 is occupied by the aromatic fluorophenyl group of KPT-9274. The second solvent exposed binding site, which is only covered by KPT-9274, consists of tyrosine 188 and tyrosine 240 that interact with the difluoro-1-piperidinyl carbonyl phenyl group via Van der Waals interactions and a hydrogen bond between the carbonyl group and the amino group of lysine 216. Importantly, the sgRNAs identified from our screen are targeting close to residues (tyrosine 18, tyrosine 240, serine 241, serine 275, and alanine 379) that are confirmed by this co-crystal to take part in the binding mode of the drug, demonstrating the power of the CRISPR-Cas9-induced mutagenesis scanning approach.

To investigate the influence of the mutations on the NAMPT structure and KPT-9274 binding, the G383del and the P236_V237del mutations were modeled utilizing MOE (The Chemical Computing Group, Montreal, Canada) implemented with the Amber99 forcefield^31^ (Fig. 4b right panels). Deletion of G383 causes a conformational change of the G383–385 stretch, which destablizes the conformation of F193 and thereby prevents pi-stacking with the ligand. Both P236_V237del and P238_Y240insAAEHS mutants cause a displacement of the loop containing the Y240 residue resulting in steric incompatibility with the KPT-9274 4,4-difluoro-1-piperidinylcarbonylphenyl group.

Altogether, these findings clearly pinpoint NAMPT as the primary target of KPT-9274 and illustrate the importance of target verification by discovery of drug resistance mutations in a cellular context.

### AsCpf1 extends the resolution of the methodology

**Fig. 5:**
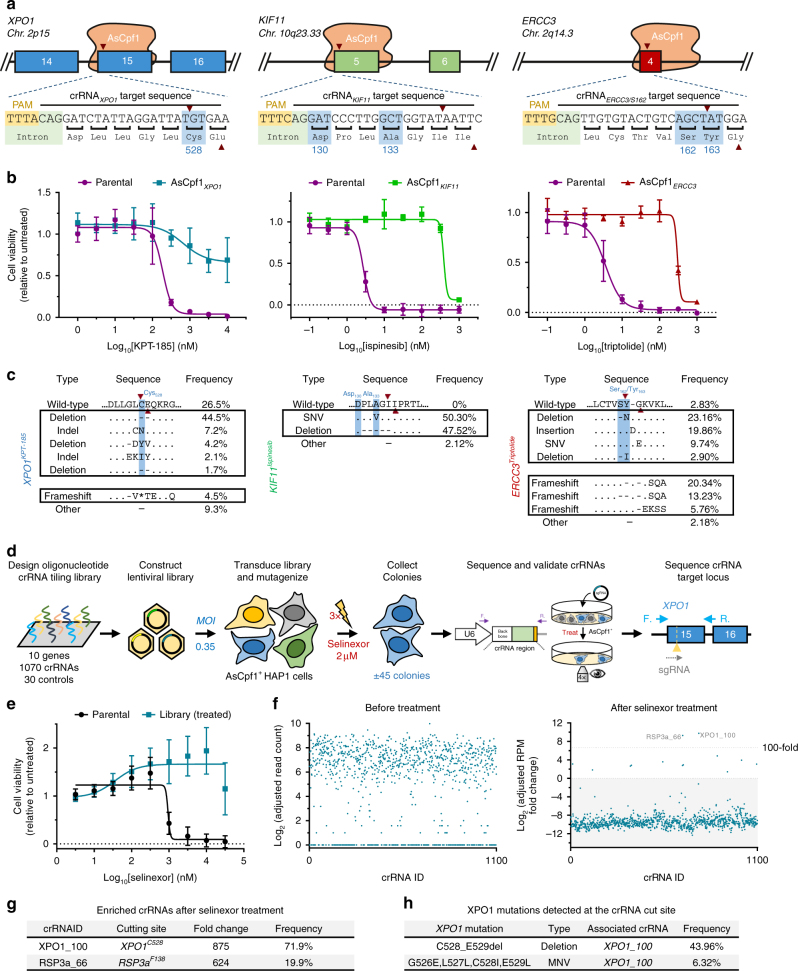
Selection of resistance mutations generated using the AsCpf1 endonuclease. **a** An overview of AsCpf1 crRNAs targeting *XPO1*, *KIF11*, and *ERCC3* at their resistance hotspot residues. The AsCpf1 cleavage site, generating a four nucleotide overhang, is denoted by red arrowheads. **b** Cell viability of wild-type and polyclonal mutagenized resistant cells in the presence of increasing concentration of drug. Values are shown relative to untreated controls. Data points represent mean ± s.d. obtained from two experiments performed in triplicate. **c** Amino-acid sequence variants determined by targeted amplicon-sequencing analysis of the resistant cells with CrispRVariants. Only variants with a frequency ≥1% are shown. **d** An overview of the CRISPR-AsCpf1 tiling crRNA library approach used for target identification of selinexor. A lentiviral tilling library targeting 10 different genes was constructed and transduced in cells stably expressing AsCpf1, which were first selected with puromycin and subsequently treated with selinexor (2 µM). crRNAs present in resistant colonies were identified by next-generation sequencing after which the genomic locus targeted by these crRNAs was sequenced to identify the resistance conferring mutations in the target gene. **e** Cell viability in the presence of different concentrations of selinexor of wild-type and resistant cells obtained after library transduction and selinexor treatment. Three experiments were performed in triplicate. Data points represent mean ± s.d. **f** Representation of the different crRNAs present in the cells before (after puromycin selection) and after treatment with selinexor as determined by EdgeR analysis of next-generation sequencing data. A value of 1 was added to each read count to facilitate log transformation. Each dot represents a different crRNA. **g** crRNA hits enriched over 100-fold in the selinexor-resistant cell pool after AsCpf1-mediated mutagenesis screen. Read counts were determined using EdgeR. **h** Amino-acid variants detected in the *XPO1* C528 locus of the resistant cells. Alterations were uncovered by CrispRVariants analysis of next-generation sequencing data

The SpCas9 mutagenesis scanning approach may be limited by the availability of NGG PAMs at or nearby the resistance hotspot of the investigated drug. To mitigate this restraint, and to orthogonally validate the strategy, we investigated whether the approach is compatible with other endonucleases recognizing a different PAM sequence such as the class 2 type V CRISPR-Cas AsCpf1^32^. We selected AsCpf1 crRNAs targeting TTTN motifs around the same codons for *XPO1, KIF11*, and *ERCC3* as described above for SpCas9 (Fig. 5a and Supplementary Fig. 13a). These were transfected along with AsCpf1 in HAP1 cells that were subsequently treated with the respective compounds. Colonies that formed within a few days of treatment were drug resistant to the specific treatment (Fig. 5b and Supplementary Fig. 13b) and contained mutations at the known hotspots (Fig. 5c and Supplementary Fig. 13c). We next designed a lentiviral tiling crRNA library, similar to the SpCas9 library, spanning all possible TTTN PAM sites in the coding sequence from 10 genes (1,100 crRNAs, Supplementary Data 8) and applied it to selinexor (Fig. 5d). Colonies rapidly appeared and were resistant to selinexor (Fig. 5e). One crRNA targeting codon C528 in *XPO1*, the anchor point of selinexor, was highly enriched (71.9%) in the resistant cell pool (Fig. 5f, g, and Supplementary Data 9). Next-generation sequencing of the *XPO1* locus revealed protein variants that mainly consisted of a deletion of residues C528 and E529 (Fig. 5h), identical to what we observed for the single crRNA (Fig. 5c). Another crRNA targeting *RPS3a* was also enriched, but could not be validated when transfected individually (data not shown). In addition, single-cell-derived clones from the original resistant cell pool revealed that this *RPS3a* crRNA always co-appeared with the *XPO1*^*C528*^ crRNA (Supplementary Fig. 14a, b), suggesting that these cells were transduced with two lentiviral particles and that decreasing the multiplicity of infection (MOI) will reduce the false-positive rate. To conclude, these results demonstrate that the CRISPR-based mutagenesis scanning approach is compatible with AsCpf1 endonuclease, showing that the approach can be tailored to other CRISPR-Cas endonucleases to increase the resolution of the method.

## Discussion

One of the major challenges in drug discovery is the identification of the cellular target of a valuable bioactive small molecule. Currently a wide range of target deconvolution technologies is available^2–4^, but the gold standard for target confirmation is the identification of mutations that confer drug resistance. Knowledge of resistance mutations is not only used as a proof for target engagement but can also provide functional insight into the molecular biology of the target protein, and can be used to rescue phenotypes from chemical genetic screens^33^.

Here, we show that genetic variation introduced by targeted CRISPR-Cas-mediated NHEJ facilitates rapid selection of in-frame drug resistance mutations in cancer cells. Because this approach exploits the cellular error-prone DNA double strand break repair mechanism, it generates a wide diversity of heterogeneous mutations. We demonstrate that this simple and fast method can be applied as a genetic screening strategy using large tiling libraries to identify the direct cellular target protein of a small molecule. An important strength of this targeted mutagenesis scanning method is that the sgRNA sequences directly annotate the genomic sequence containing the drug resistance-conferring mutations, avoiding the need for large whole-exome sequencing and complex deconvolution endeavors to uncover the relevant resistance mutations. In addition, a replicate screen allowed for the detection of extremely low-frequency (0.02%) sgRNA hits with high confidence (Fig. 3d, e) facilitating the identification of unfrequent mutational hotspots conferring drug resistance. Nevertheless, a single screen is sufficient to detect the important drug resistance-conferring sgRNAs with associated mutations (Fig. 2). However, some false-positive sgRNAs were detected from the single screen. This can be explained by co-transduction of the resistance-conferring sgRNA with another sgRNA in a small fraction of the cells, happening even at low multiplicity of infection. Another source of noise can arise from sgRNAs that were transduced in cells that already contain resistance conferring mutations  due to the heterogeneity and genetic instability of the cancer cell line. These false-positive hits can be eliminated by a replicate screen or by a simple validation experiment of transfecting individual sgRNAs (Fig. 2e). Although, in our screens, we did not observe activating mutations in other proteins besides the target protein, these may obscure the identification of the direct target itself. However, it remains to be seen how often such mutations will occur and how they affect the methodology.

Resistant cells elicited by this CRISPR-Cas mutagenesis approach were commonly hemizygous for the resistance mutation (Supplementary Fig. 1b); therefore, the approach allows for the discovery of recessive mutations avoiding the need for haploid cells. This is illustrated by triptolide resistance, for which a wild-type allele is sufficient for sensitivity^14^, in multiploid HCT 116 and HL-60 cells (Supplementary Fig. 6 and 7).

Recently, the targeting of cytidine deaminases with nuclease-deficient Cas9 has been shown to induce site-specific mutagenesis without introducing a DSB to obtain drug resistance^25,34^. The experimental set up of the dCas9-cytidine deaminase fusion approaches is different from the NHEJ-based mutagenesis approach described here but the mutational spectra overlap between both systems. However, the dCas9-cytidine deaminase fusion approaches can logically not derive deletions or insertions. We observed that in-frame deletions provide a major mechanism for drug resistance, even in functionally important protein domains. In addition, base-editing approaches are less probable to recover recessive drug resistance mutations as this requires the same mutation to be present in both alleles. Although the base editing techniques can cover a mutational hotspot region of about 100 bases, they are limited to the introduction of an average of 1.32 base substitutions per read^25^. The NHEJ-based approach described here covers a smaller hotspot region but generates larger regions of genetic variation of up to 17 bases per read.

Using the CRISPRres approach, we revealed NAMPT as the key cellular target of the clinical inhibitor KPT-9274 by showing that mutations in NAMPT confer resistance to KPT-9274. Tumor cells have been shown to be more dependent on the NAD^+^ salvage pathway catalyzed by intracellular NAMPT^35^. Overexpression of NAMPT has been observed in different cancer types and has been implicated to confer resistance to classic chemotherapeutics, whereas inhibition sensitizes cancer cells to oxidative stress and DNA-damaging agents^36^. Thus, inhibition of NAD+ biosynthesis facilitated by NAMPT shows potential as an anticancer therapy. From this perspective, multiple efforts have led to the discovery of NAMPT inhibitors^37,38^. These inhibitors induce delayed apoptosis or autophagy by gradual NAD^+^ or ATP depletion, loss of plasma membrane potential, and possibly by lowered activity of PARPs and SIRTs. Although NAMPT inhibitors have entered clinical trials as a single agent (FK866/APO866 and GMX1777/GMX1778), these failed to progress to phase 2 due to on-target dose-limiting toxicity (mainly thrombocytopenia) and lack of clinical efficacy^39^. This may partially be explained by the still poorly understood aspects of NAMPT biology in bigger biological systems, which might have led to the selection of inappropriate indications or patient populations. In addition, some of these inhibitors have relatively poor pharmacokinetics. KPT-9274 represents a new class of NAMPT inhibitors and shows promising pharmacokinetics (Karyopharm Therapeutics). Many studies have shown that KPT-9274 has promising anticancer activity in vitro and in preclinical models, and it has entered phase I clinical trials (ClinicalTrials.gov: NCT02702492). These trials are designed to include an arm with co-treatment of nicotinic acid and KPT-9274 to mitigate possible side effects due to NAMPT inhibition. The existence of a secondary, NAPRT1-dependent, NAD+ salvage pathway, which converts nicotinic acid to NAD^+^, may mitigate the observed dose-limiting toxicity. Indeed, unlike many tumor types, normal tissue cells express functional NAPRT1 and can generate NAD^+^ from nicotinic acid. Therefore, this co-treatment may be one of the keys to the success of KPT-9274 in the clinic.

Altogether our findings demonstrate that the localized genetic variation generated by CRISPR-mediated NHEJ repair can be exploited to screen essential genes for gain-of-function mutations. We establish this mutagenesis scanning approach as a genetic screen to identify the molecular target of chemical compounds inhibiting essential proteins and the method is therefore complementary to the loss-of-function screens. We envisage that this genetic screen can either be applied on a list of candidate genes identified after a first round of target deconvolution by e.g., affinity chemical proteomics, overexpression screens, or comparative studies such as CMap^40^, or it can be applied on a predefined shortlist of targets of interest. Indeed, it allows to rapidly select from a primary screen those hit molecules that target a protein or pathway of interest. Nevertheless, even when absolutely no a priori knowledge on a potential target of a hit molecule is available, the current format of the method allows coverage of all essential genes^41^ with 20–25 tiling libraries for gene–drug interaction discovery. Finally, we have illustrated the application of this genetic screen for the identification of drug–target interactions using cellular toxicity as phenotypic selection, but it may also be applicable to other phenotypic reporter assays.

## Methods

### Cell culture

HAP1 cells were obtained from Horizon Discovery. HCT 116, MIA PaCa2, and K-562 cells were obtained from ATCC, HL-60 cells from Sigma Aldrich, and OPM-2 from DSMZ. SpCas9 and AsCpf1 expressing cells were generated in house. HAP1, HL-60, and K-562 cells were grown and passaged every 2–3 days in IMDM. HCT 116 cells were grown in McCoy’s 5A medium, MIA PaCa2 cells were cultured in DMEM, and OPM-2 cells in RPMI 1640. All media were supplemented with 10% fetal bovine serum and 20 µg/mL gentamicin. Cells were incubated at 37 °C and 5% CO_2_ and were regularly checked for mycoplasma contamination using the Venor GeM OneStep PCR kit (Minerva Biolabs).

### Compounds

KPT-185, selinexor (KPT-330), KPT-9058, and KPT-9274 were provided by Karyopharm Therapeutics (Newton, MA). Ispinesib, triptolide, bortezomib, β-nicotinamide mononucleotide, and FK866 were obtained from SelleckChem. All compounds were dissolved in DMSO, except for FK866, which was dissolved in ethanol.

### DNA constructs

The plasmid expressing humanized SpCas9 was obtained from Labomics. The plasmid expressing humanized AsCpf1 was obtained from Addgene (69982). Plasmids containing sgRNAs or crRNAs were cloned in house. The pLCKO vector used for generation of the lentiviral library was obtained from Addgene (73311). The plasmid encoding for the XPO1 reporter cargo, NLS_SV40_-AcGFP-NES_PKI_, was cloned in house. Single-stranded DNA oligonucleotides for use with HDR were obtained from Integrated DNA Technologies. See Supplementary Data 10 for the sequences of the sgRNAs, crRNAs, and HDR templates.

### Generation of stable SpCas9 and AsCpf1 HAP1 cell lines

Knock-in HAP1 cell lines stably expressing SpCas9 or AsCpf1 were generated using the CRISPaint principle^42^. Briefly, cells were electroporated with the Neon Electroporation System (Thermo Fisher Scientific) using 1450 V and 3 pulses of 10 ms in 10 µL Buffer R (Neon Electroporation Kit). Cells were transfected with a plasmid encoding an sgRNA targeting the C-terminus of *SDHA* (250 ng), a plasmid encoding SpCas9 and an sgRNA targeting the donor plasmid (250 ng), and a repair donor plasmid containing a PAM and sgRNA targeting site and the sequence for P2A-mCherry-T2A-SpCas9-P2A-HygroR or T2A-AsCpf1-P2A-HyrgoR (250 ng) to stably integrate SpCas9 or AsCpf1 downstream of the *SDHA* housekeeping gene. K-562 cells were transfected with a plasmid encoding a sgRNA targeting the *AAVS1* safe harbor locus, a plasmid encoding SpCas9 and a sgRNA targeting the donor plasmid and a repair donor plasmid containing a PAM and sgRNA targeting site and the sequence for CMV-mCherry-T2A-SpCas9-P2A-HygroR to stably integrate SpCas9 in the *AAVS1* safe harbor locus. All cells were plated in a 6-well plate after transfection and 2 days later, cells were selected with 300 μg/mL hygromycin B for a period of 10 days and then checked for the expression of red fluorescent mCherry. SpCas9 or AsCpf1 endonuclease activity was assessed in the polyclonal mixtures by indel detection using TIDE^43^ in *XPO1* after sgRNA/crRNA transfection in the respective cell line.

### Transfection

Cells were transfected with the Neon Electroporation System after resuspension in Buffer R. DNA plasmids expressing the SpCas9 or AsCpf1 endonuclease and guiding RNA were added at a concentration of 37.5 ng/µL per plasmid and electroporated at 1400–1475 V with 3 pulses of 10 ms. Two to three days after transfection, cells were treated with the respective drug to select for resistance over 7–14 days. Surviving cells were counted using trypan blue exclusion staining or imaged with an IncuCyte© ZOOM (Essen Bioscience).

For HDR, a 123–134-bases-long ssDNA oligonucleotide (850 ng) was added to the electroporation mixture in addition to the respective sgRNA and SpCas9 expressing plasmids. Two days after electroporation, cells were treated with the respective drugs for 5 days before imaging with an IncuCyte© ZOOM (Essen Bioscience). Following imaging, the HDR-template transfected cells were grown under drug selection for an additional week before any further experiments were performed.

### Cell viability assays

Cell viability assays were performed by plating 3000 HAP1, 5000 HCT 116, K-562, or HL-60 cells in 96-well plates containing a dilution of the test compound. Cells were incubated for 72 h at 37 °C and 5% CO_2_. Cell viability was then assessed with the CellTiter 96® AQueous Non-Radioactive Cell Proliferation Assay (Promega) and colorimetric signals were measured with a Safire2™ (TECAN). Assays were performed in triplicate and each experiment was repeated at least once. Obtained values were adjusted with the background signal and divided by the untreated control. Relative data values were then visualized and analyzed using a log-based 4 parameter model (GraphPad Prism).

For single-guide drug resistance validation assays, 125.000 MIA PaCa2, OPM-2, or HAP1/SpCas9+ and K-562/pCas9^+^ cells were transfected with plasmids expressing individual sgRNAs, and if needed SpCas9, using the Neon Electroporation system as described above and plated into 6-well plates. Cells were treated 2–3 days after transfection with the respective compound for a period of 5–7 days, the medium was regularly refreshed, and dead cells were washed away before imaging confluency using a live cell analysis system (Essen Bioscience, IncuCyte ZOOM®).

### DNA extraction and sequencing

Genomic DNA was isolated from 1 million cells with the QIAamp DNA mini kit using RNase A. For Sanger sequencing, the region of interest was amplified by PCR with the CloneAmp HiFi PCR premix (Clontech). The amplified DNA was then purified (QIAquick PCR purification kit (Qiagen)) and sequenced (Macrogen). For targeted amplicon next-generation sequencing of single-guide transfected cells, the region of interest (*KIF11*_*A133*_,* XPO1*_*C528*_,* ERCC3*_*D54*_, *ERCC3*_*S162*_,* NAMPT*_*Y18*_,* NAMPT*_*S240*_, or *NAMPT*_*G383*_) was first amplified over 24 cycles in 25 µL PCR reactions containing 50 ng genomic DNA with the Phusion® High-Fidelity PCR Master Mix with HF Buffer (NEB) and with custom primers containing adapter regions for Nextera indexes (IDT). Amplified DNA was purified with the QiaQuick PCR purification kit (Qiagen) and 1.5–2 µL of this DNA was PCR amplified over 25 cycles with CloneAmp HiFi PCR Premix (Clontech) using indexing primers containing P5 and P7 Illumina adapters in 25 µL reactions to index the samples. Indexed samples were purified using magnetic Agencourt AMPure XP beads (Beckman Coulter) and eluted in TE buffer. Samples were then diluted to 2–4 nM and pooled to form the initial library. This library was then denatured and diluted according to the instructions for paired-end sequencing on a MiSeq (Illumina) with a MiSeq V2–300 or 500 cycles kit (Illumina) and 10% PhiX v3 (Illumina) spike-in. For a list of primers see Supplementary Data 10.

### Analysis of next-generation sequencing data

FastQ files obtained after MiSeq sequencing were demultiplexed with the MiSeq Reporter software (Illumina). Demultiplexed and paired reads were trimmed, filtered, and then aligned to the reference amplicon in Geneious (v9, Biomatters). To obtain haplotypes present in drug-resistant samples, bam files were analyzed with the CrispRVariants package run in RStudio by defining a 35–140 bp spanning region across the endonuclease cut site, as defined by pre-analysis of localized variants within Geneious. For this purpose, the CrispRVariants “readtotarget” input was run with parameters “upstream.snv” (15–70) and “downstream.snv” (15–70) on corresponding paired end sequencing reads to allow for haplotype determination. Haplotype nucleotide sequences were extracted with a small script. Nucleotide sequences were then visualized and mapped to the reference in Geneious v9 (Biomatters) and amino-acid variants were determined. For visualization of the spectra of single-nucleotide variants, NGS reads were aligned to the reference gene. Nucleotide occurrence frequencies were then determined in R on the aligned NGS reads using the deepSNV Bioconductor package. Sequences containing sgRNA/crRNAs from the pooled lentiviral screens were trimmed from adapter sequences. Individual sgRNA/crRNA sequencing reads were then counted with EdgeR^44^ and the fold change of enriched sgRNAs was calculated as follows and visualized using GraphPad Prism:

$${\mathrm{Log}}_{\mathrm{2}}\left( {\frac{{1 + ({\mathrm{Read}}\,{\mathrm{Count}}\,{\mathrm{per}}\,{\mathrm{Million}}\,{\mathrm{Reads}})_{{\mathrm{after}}\,{\mathrm{drug}}\,{\mathrm{treatment}}}}}{{1 + ({\mathrm{Read}}\,{\mathrm{Count}}\,{\mathrm{per}}\,{\mathrm{Million}}\,{\mathrm{Reads}})_{{\mathrm{after}}\,{\mathrm{puromycin}}\,{\mathrm{selection}}}}}} \right).$$

### Cloning of the sgRNA and crRNA libraries

The 2,209 sgRNA sequences used in the ispinesib-KIF11 pilot screen were obtained by selecting all N_21_GG sequences available in the NCBI consensus coding sequences of the main isoforms of 9 genes (*KIF11*,* XPO1*,* ERCC3*,* PAK4*,* ABL1*,* TUBB*,* ACTB*,* RPS3a*, and *H2BFM2*). Also included were 100 control sgRNAs and all sgRNA sequences were appended 5′ and 3′ with small DNA sequences to facilitate PCR (total length 60 nt).

To obtain the AsCpf1 crRNA sequences, the coding sequence of the main isoforms of 10 genes (*KIF11*,* XPO1*,* ERCC3*,* PAK4*,* ABL1*,* TUBB*,* ACTB*,* RPS3a*,* H2BFM2*, and *p53*) were extracted from NCBI. For each intron–exon boundary, 25 nucleotides were added to the exonic sequences. From these sequences, all TTTN_24_ sequences were extracted and appended 5′ with the AsCpf1 direct repeat backbone (TAATTTCTACTCTTGTAGA) and 30 scrambled control crRNAs were included. Sequences were then further appended 5′ and 3′ with small DNA sequences to facilitate PCR (total length 79 nt).

The sgRNA sequences for the “FDA-target” and “non-FDA-target” libraries were obtained with a custom script run in RStudio. In brief, target genes for the libraries were roughly determined by the drug target list available for approved and investigational antineoplastic agents on the Kyoto Encyclopedia of Genes and Genomes (KEGG) database (retrieved July 2016) combined with a small literature study. The target list was then filtered from agents consisting of analogs of nucleotide or metabolic products. The web-based Biomart (Ensemble) was used to obtain the start and end coordinates of all CDS exons retrieved from the NCBI refseq entries available for all isoforms of the predefined target genes. Twenty base pairs were added 5′ and 9 bp were added 3′ to each of the exonic start or end coordinates on the forward and reverse strands, respectively, to include sgRNAs located on exon–intron boundaries. These expanded and strand-specific coordinates were used to search through the NCBI reference sequences to obtain all N_21_GG sequences within these coordinates on both the forward and reverse strands. Duplicate sgRNA sequences were removed on a gene-per-gene basis, sgRNAs containing a TTTTT sequence were removed and sgRNAs were then appended with additional sequences to facilitate PCR and the generation of subpools. See Supplementary Data 1, 3, 4, and 8 for the gene target lists and the individual sgRNA sequences.

All appended sgRNA and crRNA sequences were synthesized as pools by Customarray Inc. (Bothell, WA) on a 12K (9/10 gene libraries) or 90K (“non-FDA-target”/”FDA-target”) chip. The sgRNA/crRNA pools were amplified in 10 parallel reactions (25 μL, 1 ng input) by PCR with the CloneAmp HiFi premix kit (Clontech) and PCR products were purified with the QIAQuick Nucleotide Removal kit (QiaGen). The purified PCR products were then subjected to restriction digestion (six parallel reactions) with BfuAI (NEB) overnight at 50 °C. After digestion, six ligation reactions containing 33 ng of digested sgRNA/crRNAs and 500 ng of the BfuAI and NsiI predigested pLCKO vector were performed overnight at 16 °C with T4 DNA ligase (NEB). The pooled mixture of ligated pLCKO vectors was then purified with the QIAquick nucleotide removal kit (Qiagen) and electroporated into Endura competent cells (Lucigen) with a Gene Pulser system (Biorad) according to the manufacturer’s instructions. Transformed cells were then plated in 15-cm-diameter petridishes containing prewarmed LB agar with 100 µg/mL ampicilin and grown overnight at 32 °C. The following day colonies were counted and a fold representation of 400 (“FDA” libraries A and B), 2700 (“non-FDA” libraries C and D), 30,000 (9 gene Cas9 library), or 90,000 (AsCpf1 library) was estimated. All colonies were pooled per library for plasmid extraction with the PureLink® HiPure Plasmid Maxiprep (Invitrogen).

### Lentiviral library

The pooled and purified pLCKO-U6-sgRNA/crRNA plasmid libraries were provided to Applied Biological Materials Inc. (Richmond, BC, Canada) to generate lentiviral particles coated with the VSV-G protein and containing the desired genetic information for human expression of the sgRNA/crRNAs. Viral stocks were titrated on wild-type HAP1 or K-562 cells to determine the MOI.

### Target identification screens

HAP1 and K-562 cells stably expressing SpCas9 or AsCpf1 were resuspended in supplemented IMDM containing 8 µg/mL polybrene and transduced with lentiviral particles containing the desired sgRNA/crRNAs at an MOI of 0.25 (coverage of 5000× per sgRNA for SpCas9) or 0.35 (coverage of 15,000× per crRNA for AsCpf1) by spinfection in 12-well plates containing 2 × 10^6^ cells/well. For the bortezomib resistance screen, ~300 million cells were transduced with sublibrary B, while for the KPT-9274 resistance screen ~400 million were transduced with both sublibraries C and D. The next day cells were transferred to T150 cell flasks and were allowed to develop mutations for a period of 3–5 days under puromycin selection. Then 4 million (ispinesib/selinexor) or 10 million (KPT-9274/bortezomib) cells were harvested for DNA extraction and the remaining cells were treated for a period of 2–3 weeks with 8 nM ispinesib, 30 nM bortezomib, 300 nM (HAP1) or 500 nM (K-562) KPT-9274, or 2 µM selinexor. The compound-containing medium was refreshed three times. After treatment, surviving cells were harvested and the genomic DNA was extracted using the QiaGen DNA mini kit and subjected to 5–10 parallel 100 µL PCR reactions (24 cycles, 2000 ng per reaction) with pLCKO primers carrying Nextera adapter sequences and using the Phusion High-Fidelity PCR mastermix with HF buffer (NEB). Amplified DNA was purified and pooled and a second PCR using the CloneAmp HiFi Polymerase kit (Clontech) was performed over 24 cycles on 50 ng with Nextera indexing primers (Illumina). Further processing for next-generation sequencing analysis was performed as described above.

### XPO1-mediated nuclear export phenotypic reporter assay

To study the XPO1-mediated nuclear export, HAP1/SpCas9^+^ cells were transfected with a plasmid encoding for the NLS-AcGFP-NES reporter construct and a plasmid pool encoding 4 sgRNAs targeting *XPO1* for knockout. Cells were transfected with the Neon Electroporation system as described above and plated in µ-slide 8-well glass bottom plates (IBIDI). Two days after transfection, cells were treated with DMSO or 1 µM selinexor for 3 h. After incubation, the AcGFP reporter construct was visualized in live cells using a Leica SP5 confocal microscope employing a CX PL APO 63x (NA 1.2) objective. AcGFP was excited at 488 nm (argon laser) and emission was detected at 492–560 nm.

### Pull-down of XPO1 with KPT-9058

For pull-down of XPO1, parental HAP1 and HDR-edited HAP1 cells were incubated with 2 µM of KPT-9058 for 3 h. Following treatment, cells were washed in ice-cold PBS and pellets were lysed on ice in RIPA buffer supplemented with 1× HALT protease inhibitors (Thermo Scientific). Samples were cleared from debris by centrifugation at 18,000×*g* for 10 min at 4 °C. Protein concentrations were measured with a colorimetric BSA protein assay (Pierce). A fraction of the cell lysis mixture was taken for quantification of β-tubulin and XPO1 total protein. Remaining extracts were incubated with Dynabeads MyOne Streptavidin T1 (Life Technologies) to capture XPO1-bound KPT-9058 by rotating overnight at 4 °C. The next morning, beads were collected with the DnyaMag-2 (Life Technologies) and washed five times in modified RIPA buffer (50 mM Tris-HCl pH 7.8, 150 mM NaCl, 1% NP-40 (IGEPAL CA-630), 0.1% sodium deoxycholate, 1 mM EDTA). The captured proteins were eluted by boiling the samples for 10 min in 0.5% SDS containing 1× sample buffer (Protein Simple). Samples were separated by size (12–230 kDa) and visualized on a Wes system (Protein Simple) with an anti-rabbit HRP conjugated antibody detecting the primary XPO1 (1/12,500, NB100–79,802) and β-Tubulin (1/3000, NB600–936) antibodies. Protein signals were visualized and quantified with the Compass software, v2.7.1 (Protein Simple).

### Immunofluorescence analysis of mitotic spindle formation

Parental HAP1 and HDR-edited HAP1 cells were electroporated with a plasmid pool encoding for four sgRNAs targeting *KIF11* (for KIF11 knockout) or the *AAVS1* locus (control) and plated in 8-well chamber glass slides. The next morning, cells were treated with DMSO or 50 nM ispinesib for 4 h. Cells were fixed for 10 min at room temperature using 4% PFA and permeabilized with PBS containing 0.1% Triton X-100 for 10 min. Afterwards, cells were washed with PBS and blocked with 10% normal goat serum for 1 h at 37 °C. Subsequently, cells were stained with an α-tubulin primary antibody (sc5286, Santa Cruz Biotech) dissolved in 10% normal goat serum for 1 h at 37 °C. Following primary staining, cells were washed two times with PBS and stained with a secondary antibody conjugated to Alexa Fluor 488 (1/500, A-11001, Invitrogen) in the dark for 45 min at 37 °C. Nuclei were counterstained using the NucBlue fixed Cell stain (Life Technologies). Stained cells were imaged using a Leica SP5 confocal microscope employing a CX PL APO 63× (NA 1.2) water immersion objective. Alexa Fluor 488 and DAPI were detected using the excitation line of 488 nm (argon laser) or the excitation line of 405 nm (pulsed diode laser), respectively. Blue emission was detected between 410 and 480 nm, and green emission was detected between 492 and 560 nm.

### Determination of proteasome activity

Proteasome activity was determined using the Proteasome Glo^TM^ Assay (Promega), and the assay was performed according to the manufacturer’s instructions. Briefly, site-specific luminogenic substrates were added to determine the activity of the different catalytically active sites (Suc-LLVY-aminoluciferin). Proteasome activity was determined at baseline and after a 2-h incubation period (at 37 °C, 5% CO_2_) with 12.5 nM of bortezomib. The read-out was performed on a Wallac Victor plate reader.

### Determination of NAD+ levels

HAP-1 cells were plated in 96-well plates and incubated overnight. The next day, a dilution series of KPT-9274 was added and incubated overnight. NAD^+^ levels were determined the following day using luminescence and the NAD+/NADH Glo Assay (Promega). NAD^+^ was obtained from SelleckChem to generate a standard curve.

### NAMPT crystalization

NAMPT was purified as previously described^29^. Briefly, full-length human NMPRTase was expressed with a C-terminal His tag in *Escherichia coli* at 20 °C. A combination of nickel-agarose affinity chromatography, anion exchange and gel-filtration chromatography was used to purify the protein. Crystals were grown for 5 days at 20 °C in hanging drops with a reservoir solution of 0.2 M NaCl, 0.1 M Tris pH 8.5, and 22% PEG 3350. The drops were composed of 1 μL NAMPT protein (10 mg/mL) and a 5-fold excess of KPT-9274 in a 25 mM Tris, 150 mM NaCl, 5 mMM DTT pH 8.0 buffer and 1 μL of reservoir solution. Crystals were shortly soaked in a cryoprotectant consisting of the reservoir solution with an additional 10% glycerol and flash-frozen in liquid nitrogen. Diffraction data were collected at the SSRF-BL17B beamline at 100 K using 0.95369 Å radiation using a Rayonix MX300 detector and processed with hkl3000. PDB entry 4WQ6 was used as a starting point for molecular replacement and structure refinement utilizing CCP4^45^, Phenix^46^, and Coot^47^ (see Supplementary Fig. 12). The KPT-9247 structure was built into the difference map at the ligand bind site, which shows a good agreement with the electron density (see Supplementary Fig. 12). Following introduction of the mutations by modeling utilizing MOE, the conformation of the residues within a 10-Å radius of the ligand was energy optimized and the conformation changes at the ligand-binding site were observed.

### Data availability

The coordinates of the crystal are submitted to wwPDB as 5NSD. The used R scripts can be found in the Supplementary Software file. Sequencing data are available from the NCBI Sequencing Read Archive under the accession code SRP126384 (Bioproject PRJNA419880). All other data are available from the authors upon request.

## Electronic supplementary material

Supplementary Information

Description of Additional Supplementary Files

Supplementary Data 1

Supplementary Data 2

Supplementary Data 3

Supplementary Data 4

Supplementary Data 5

Supplementary Data 6

Supplementary Data 7

Supplementary Data 8

Supplementary Data 9

Supplementary Data 10

Supplementary Data 11
