# Supplementary material for: Target identification of small molecules using large-scale CRISPR-Cas mutagenesis scanning of essential genes
Source: Nat Commun. 2018 Feb 5;9:502. doi: 10.1038/s41467-017-02349-8 (PMC5799254; doi:10.1038/s41467-017-02349-8)
Supplement: Supplementary file 1 — Supplementary Information [file 41467_2017_2349_MOESM1_ESM.pdf]

Supplementary Figure 1

a

Selection (KPT-185)	Type	Protein Sequence	Read Frequency	CrispRVariants Identifier
300 nM	Wild-type	...LGLCEQKRGKD...	1.9%	No variant
300 nM	MNV	...SVHI....	28.8%	SNV: -10,-7,-6,-3,-1
300 nM	Indel	..YVNR.....	4.2%	-14:1D,-2:1I
300 nM	Deletion	...----....	1.5%	-12:12D
300 nM	Deletion	..-DPPV....	1.2%	-14:1D,-10:1D,-2:1D
300 nM	Frameshift	........KRQR	27.1%	-2:1I
300 nM	Frameshift	........EAKI	5.8%	-2:1D
300 nM	Frameshift	......--RQR	4.4%	-4:5D
300 nM	Other	-	25.1%	Other
600 nM	Wild-type	...LGLCEQKRGKD...	0.9%	No variant
600 nM	Indel	..YVNR.....	36.8%	-14:1D,-2:1I
600 nM	Indel	..SIRII....	5.1%	-13:1I,-5:1D
600 nM	Deletion	.DYVNR--...	2.7%	-17:1D,-4:5D
600 nM	Deletion	...--SI....	1.3%	-10:5D,-4:1D
600 nM	Deletion	...GL--....	1.3%	-11:1D,-9:1D,-6:4D
600 nM	Frameshift	........EAKI	37.9%	-2:1D
600 nM	Other	-	14.0%	Other
1500 nM	Wild-type	...LGLCEQKRGKD...	1,1%	no variant
1500 nM	Deletion	...--K.....	59,1%	-11:4D,-5:1D,-3:1D
1500 nM	Deletion	...--......	7,3%	-12:6D
1500 nM	Indel	...ILIL....	4,6%	-12:1I,-3:1D
1500 nM	MNV	...SVHII...	2,6%	SNV:-10,-7,-6,-3,-1,3
1500 nM	Other	-	25,3%	Other
2000 nM	Wild-type	...LGLCEQKRG-KD...	1.1%	No variant
2000 nM	Deletion	..-YVNR.....	27.5%	-14:1D,-2:2D
2000 nM	Deletion	..-KDNI.....	10.1%	-15:2D,-11:1D
2000 nM	Indel	..FAIASHNS..	6.2%	-12:2I,-10:1I,-7:1I,5:1D
2000 nM	Deletion	..-SVHI.....	1.1%	-14:1D,-8:2D
2000 nM	Frameshift	........KEA.I	23.1%	-2:2I
2000 nM	Frameshift	......--R.QR	11.3%	-4:5D
2000 nM	Frameshift	......--A..I	2.4%	-4:7D
2000 nM	Other	-	17.2%	Other

Selection (KPT-185)	Type	Protein Sequence	Read Frequency	CrispRVariants Identifier
300 nM	Wild-type	...LGLCEQKRGKD...	78.0%	No variant
300 nM	Deletion	...--......	1.2%	-12:6D
300 nM	Indel	...AAWE....	1.1%	-11:1D,-4:1I
300 nM	Frameshift	........-QR	3.3%	-2:2D
300 nM	Other	-	16.4%	Other
600 nM	Wild-type	...LGLCEQKRGKD...	9.4%	No variant
600 nM	Deletion	...DR--....	12.9%	-11:1D,-9:3D,-2:2D
600 nM	Deletion	...SI--....	11.7%	-10:5D,-4:1D
600 nM	Deletion	...Q--.....	10.7%	-12:6D
600 nM	Deletion	..SV--.....	6.6%	-14:1D,-8:5D
600 nM	Deletion	..FASI-....	5.6%	-12:1D,-9:1D,-4:1D
600 nM	Indel	..FTGSI....	4.7%	-14:1I,-4:1D
600 nM	Deletion	..KDNI-....	1.7%	-15:2D,-11:1D
600 nM	MNV	...IDNN....	1.5%	SNV:-11,-10,-9,-6,-5,-3,1
600 nM	Deletion	......--N..	1.1%	1:6D
600 nM	Frameshift	......--RQR	11.6%	-4:5D
600 nM	Frameshift	........KRQR	3.0%	-2:1I
600 nM	STOP	...*---....	1.3%	-10:9D
600 nM	Other	-	18.2%	Other
1500 nM	Wild-type	...LGLCEQKRGKD...	2.4%	No variant
1500 nM	Indel	...SIRI....	21.8%	-8:1D,-3:1I
1500 nM	Deletion	..FVHI-....	8.2%	-12:1D,-8:2D
1500 nM	Indel	..SVNLLG...	6.0%	-14:1D,-5:1I
1500 nM	Deletion	...K--.....	2.2%	-11:4D,-5:1D,-3:1D
1500 nM	Deletion	..FQR--....	1.3%	-12:1D,-10:5D
1500 nM	Deletion	...GL--....	1.2%	-11:1D,-9:1D,-6:4D
1500 nM	Frameshift	.....---A.I	20.1%	-8:10D
1500 nM	Frameshift	......----I	6.9%	-4:13D
1500 nM	Frameshift	......----R	2.9%	-2:11D
1500 nM	Other	-	27.1%	Other
2000 nM	Wild-type	...LGLCEQ-KRGK...	2.5%	No variant
2000 nM	Insertion	..FASVHI...	23.1%	-12:3I
2000 nM	Indel	.CANRS.I...	12.7%	-8:1I,-4:1D
2000 nM	MNV	.CSHNP.N...	8.2%	SNV:-17,-15,-13,-11,-10,-8,-6,-4,-3,1
2000 nM	Deletion	...Q--.....	2.5%	-12:6D
2000 nM	MNV	...GKD.LL..	2.3%	SNV:-11,-9,-8,-5,-3,-2,-1,2,3
2000 nM	Insertion	......N....	1.8%	-2:3I
2000 nM	Deletion	..SYV-.-...	1.4%	-7:6D
2000 nM	Deletion	..SVHI.-...	1.2%	-14:1D,-8:2D
2000 nM	Deletion	...SI-.-...	1.2%	-10:5D,-4:1D
2000 nM	Frameshift	.......--RQ	13.8%	-4:5D
2000 nM	Other	-	29.3%	Other

b

Type	Sample (KPT-185)	Apparent Ploidy	*XPO1* Genotype	Protein
**Wild-type**	-	Haploid	ATTAGGATTATGTGAACAGAAAAGAGGC<br>–	-<br>-
**Clone 1**	300 nM	Haploid	ATTAGGATTA**GATCTATT**------AGGC<br>–	p.C528_R532delinsDLL<br>-
**Clone 2**	300 nM	Diploid	ATTAGGATTAT**C**TG**TT**CA**C**A**T**AAGAGGC<br>ATTAGGATTATGTGAACAGAAA-GAGGC	p.C528S, E529V, Q530H, K531I<br>p.R532fsX16
**Clone 3**	300 nM	Diploid	ATTAGGATTAT**C**TG**TT**CA**C**A**T**AAGAGGC<br>ATTAGGATTATGTGAACAGAAA-GAGGC	p.C528S, E529V, Q530H, K531I<br>p.R532fsX16
**Clone 4**	2,000 nM	Diploid	ATTAGGATT**TGCTATTGCCTCACATAATA**GC<br>ATTAGGATTATGTGAAC--------AGGC	p.L527_G533delinsFAIASHNS<br>p.K531fsX15
**Clone 5**	2,000 nM	Diploid	ATTAGGAT-ATGTGAACAG--AAGAGGC<br>ATTAGGATTATGTGAACAGA**AG**AAAGAGGC	p.L527_R532delinsYVNR<br>p.K531fsX17
**Clone 6**	2,000 nM	Diploid	ATTAGG--**CA-AAGATAATATT**AGAGGC<br>ATTAGGATTATGTGAAC-----AGAGGC	p.L527_L531delinsKDNI<br>p.K531fsX4

c

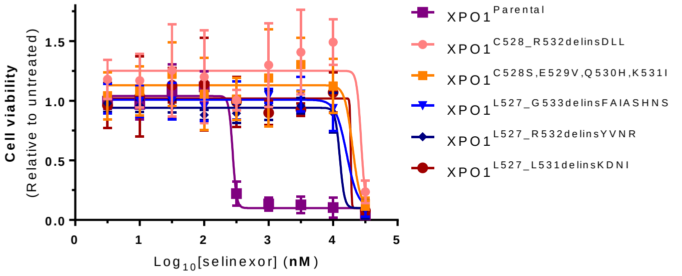

Supplementary Figure 1: protein variants present in *XPO1* genome edited cells after treatment with different concentrations of KPT-185.

a. XPO1 amino acid variants detected in HAP1 cells transfected with SpCas9 and sgRNA$_{XPO1/L531}$. Cells were treated with four different concentrations of KPT-185 and the experiment was repeated once. Haplotype nucleotide sequences were obtained by next generation sequencing analysis with CrispRVariants and translated into amino acids. Variants detected above the 1% threshold are shown. C528, the anchor point for binding by KPT-185, is highlighted in blue and the Cas9 cut site is shown with a red arrowhead and a vertical dotted line.

b. Genotypes of single-cell derived, KPT-185 resistant, *XPO1* genome edited HAP1 colonies. Single cell derived clones were obtained by plating 0.5 cells/well in 96-well plates and subjected to Sanger sequencing. In the wild-type reference sequence, the sgRNA targeting sequence is underlined, while the PAM site is highlighted in red. Bold nucleotides highlight mutations. The codon encoding the cysteine$_{528}$ residue (TGT) is highlighted in the blue column. The results confirm that some HAP1 cells had turned diploid at the XPO1 locus during the experiment.

c. Cell viability after 72h of single-cell derived clones obtained from the pool of resistant HAP1 cells in the presence of selinexor. Data points are normalized relative to untreated cells and represent means ± s.d. obtained from two experiments performed in triplicate.

Supplementary Figure 2

Selection (Ispinesib)	Type	Protein Sequence	Read Frequency	CrispRVariants Identifier
		Asp$_{130}$ ▼ Ala$_{133}$		
4 nM	Wild-type	...DPL-AGIIP...	2.3%	No variant
4 nM	Deletion	---......	51.4%	-9:9D
4 nM	Deletion	..-......	20.9%	-3:3D
4 nM	Insertion	..FL.....	2.3%	-3:3I
4 nM	Deletion	.--.-....	2.3%	-6:9D
4 nM	Deletion	..-.-....	2.0%	-4:6D
4 nM	Deletion	.-.......	1.3%	-4:3D
4 nM	Deletion	..-......	1.1%	-2:3D
4 nM	Frameshift	..F.GWYNS	1.4%	-2:1I
4 nM	Frameshift	..-.-WYNS	1.3%	-3:5D
4 nM	Stop	....L*FQA	1.0%	1:12I
4 nM	Other	–	12.7%	Other
		Asp$_{130}$ ▼ Ala$_{133}$		
8 nM	Wild-type	...DP-LAGIIP...	1.0%	No variant
8 nM	Deletion	--.-.....	53.4%	-9:9D
8 nM	Deletion	...-.....	21.1%	-3:3D
8 nM	Insertion	..F......	2.7%	-3:3I
8 nM	Deletion	...-.....	1.5%	-2:3D
8 nM	Deletion	.-.--....	1.5%	-6:9D
8 nM	Deletion	...--....	1.4%	-4:6D
8 nM	Deletion	.-.......	1.2%	-4:3D
8 nM	Stop	...L*FQAG	1.4%	1:12I
8 nM	Frameshift	...FGWYNS	1.3%	-2:1I
8 nM	Frameshift	...--WYNS	1.0%	-3:5D
8 nM	Other	–	12.5%	Other
		Asp$_{130}$ ▼ Ala$_{133}$		
20 nM	Wild-type	...DPLAGIIP...	0.0%	No variant
20 nM	Deletion	..-.....	39.5%	-3:3D
20 nM	Deletion	---.....	28.9%	-9:9D
20 nM	Deletion	...---..	1.8%	-1:9D
20 nM	Deletion	E--.....	1.7%	-6:6D
20 nM	Deletion	..--....	1.7%	-4:6D
20 nM	Deletion	..-.....	1.5%	-2:3D
20 nM	Deletion	.---....	1.3%	-6:9D
20 nM	Frameshift	..FGWYNS	3.8%	-2:1I
20 nM	Frameshift	..--WYNS	1.7%	-3:5D
20 nM	Frameshift	...GWYNS	1.5%	1:1I
20 nM	Frameshift	..-WLV*F	1.5%	-2:1D
20 nM	Frameshift	---WLV*F	1.2%	-14:13D
20 nM	Frameshift	E------F	1.2%	-7:16D
20 nM	Other	–	12.7%	Other
		Asp$_{130}$ ▼ Ala$_{133}$		
40 nM	Wild-type	...DPL-AGIIP...	0.9%	No variant
40 nM	Deletion	---......	43.6%	-9:9D
40 nM	Deletion	..-......	25.7%	-3:3D
40 nM	Deletion	.-.......	4.7%	-4:3D
40 nM	Deletion	..-.-....	2.3%	-4:6D
40 nM	Deletion	-A.......	1.5%	-7:3D
40 nM	Insertion	..FL.....	1.5%	-3:3I
40 nM	Deletion	..-......	1.2%	-5:1D,-1:2D
40 nM	Deletion	.--.-....	1.1%	-6:9D
40 nM	Deletion	E--......	1.0%	-6:6D
40 nM	Frameshift	..-FGWYNS	2.0%	-2:1I
40 nM	Frameshift	.--.---NS	1.9%	-7:14D
40 nM	Other	–	12.6%	Other

Selection (Ispinesib)	Type	Protein Sequence	Read Frequency	CrispRVariants Identifier
		Asp$_{130}$ ▼ Ala$_{133}$		
4 nM	Wild-type	...DP-LAGIIP...	1.1%	No variant
4 nM	Deletion	--.-.....	53.4%	-9:9D
4 nM	Deletion	...-.....	18.9%	-3:3D
4 nM	Deletion	.X.-.....	2.7%	-4:3D
4 nM	Insertion	..F......	2.2%	-3:3I
4 nM	Deletion	...--....	1.5%	-4:6D
4 nM	Deletion	.-.--....	1.1%	-6:9D
4 nM	Frameshift	...FGWYNS	2.8%	-2:1I
4 nM	Frameshift	....GWYNS	1.2%	1:1I
4 nM	Frameshift	...FWLV*F	1.1%	-2:2I
4 nM	Frameshift	...--WYNS	1.0%	-3:5D
4 nM	Other	–	12.9%	Other
		Asp$_{130}$ ▼ Ala$_{133}$		
8 nM	Wild-type	...DP-LAGIIP...	0.3%	No variant
8 nM	Deletion	--.-.....	54.1%	-9:9D
8 nM	Deletion	...-.....	17.9%	-3:3D
8 nM	Deletion	.X.-.....	3.7%	-4:3D
8 nM	Insertion	..F......	2.3%	-3:3I
8 nM	Deletion	.-.--....	1.6%	-6:9D
8 nM	Deletion	E-.-.....	1.3%	-6:6D
8 nM	Deletion	...--....	1.1%	-4:6D
8 nM	Frameshift	...FGWYNS	2.4%	-2:1I
8 nM	Frameshift	....GWYNS	1.4%	1:1I
8 nM	Frameshift	--.-GWYNS	1.0%	-8:8D
8 nM	Frameshift	...--WYNS	1.0%	-3:5D
8 nM	Other	–	12.0%	Other
		Asp$_{130}$ ▼ Ala$_{133}$		
20 nM	Wild-type	...DP-LAGIIP...	0.2%	No variant
20 nM	Deletion	--.-.....	50.7%	-9:9D
20 nM	Deletion	...-.....	20.5%	-3:3D
20 nM	Deletion	.X.-.....	3.3%	-4:3D
20 nM	Deletion	...--....	2.8%	-4:6D
20 nM	Insertion	..F......	2.5%	-3:3I
20 nM	Deletion	.-.--....	1.6%	-6:9D
20 nM	Deletion	E-.-.....	1.3%	-6:6D
20 nM	Frameshift	...FGWYNS	2.4%	-2:1I
20 nM	Frameshift	....GWYNS	1.8%	1:1I
20 nM	Frameshift	....-LV*F	1.6%	1:1D
20 nM	Other	–	11.3%	Other
		Asp$_{130}$ ▼ Ala$_{133}$		
40 nM	Wild-type	...DP-LAGIIP...	0.4%	No variant
40 nM	Deletion	--.-.....	38.4%	-9:9D
40 nM	Deletion	...-.....	26.9%	-3:3D
40 nM	Deletion	.-.--....	4.3%	-6:9D
40 nM	Deletion	.X.-.....	3.6%	-4:3D
40 nM	Insertion	..F......	2.9%	-3:3I
40 nM	Deletion	...-.....	2.7%	-2:3D
40 nM	Deletion	...--....	2.0%	-4:6D
40 nM	Deletion	....---..	1.0%	-1:9D
40 nM	Deletion	...-.....	1.0%	-5:1D,-1:2D
40 nM	Frameshift	...FGWYNS	2.2%	-2:1I
40 nM	Frameshift	....GWYNS	2.0%	1:1I
40 nM	Frameshift	...FWLV*F	1.0%	-2:2I
40 nM	Other	–	11.5%	Other

Supplementary Figure 2: KIF11 protein variants present in genome edited and ispinesib resistant cells.
KIF11 amino acid variants detected in HAP1 cells transfected with SpCas9 and sgRNA$_{KIF11/L132}$. Transfected cells were treated with four different concentrations of ispinesib and the experiment was repeated twice, but the results from only 2 experiments are shown. Protein variants were obtained by CrispRVariants analysis of next generation sequencing reads. Only variants detected above the 1% threshold are shown. Residues known to provide drug resistance upon mutation are highlighted in blue and the Cas9 cut site is shown with a red arrowhead and a vertical dotted line.

Supplementary Figure 3

Selection (Triptolide)	Type	Protein Sequence	Read Frequency	CrispRVariants Identifier
		Ser_{162}/Tyr_{163}		
2 nM	Wild-type	...VSYGKV-KLVL...	81.8%	No variant
2 nM	Insertion	.......L....	3.1%	-1:3I
2 nM	MNV	......D.Q...	1.1%	SNV:-3,-1
2 nM	Frameshift	......-.-AG.	2.3%	-6:5D
2 nM	Frameshift	.........AG.	2.2%	-1:1I
2 nM	Other	–	9.5%	Other
		Ser_{162}/Tyr_{163}		
4 nM	Wild-type	...VSYGKV--KLVL...	21.1%	No variant
4 nM	Insertion	........L....	24.6%	-1:3I
4 nM	Deletion	.........---.	3.9%	-4:9D
4 nM	MNV	......D..Q...	3.7%	SNV:-3,-1
4 nM	Insertion	.....AME....	2.9%	-3:1I,-2:5I
4 nM	Insertion	........XQ...	1.7%	1:3I
4 nM	Deletion	......-..-...	1.5%	-7:6D
4 nM	Frameshift	......--..AG.	8.1%	-6:5D
4 nM	Frameshift	..........AG.	7.2%	-1:1I
4 nM	Frameshift	..----..--WS	2.7%	-16:16D
4 nM	Frameshift	.........XAG.	2.7%	1:1I
4 nM	Frameshift	.........--WS	1.2%	-2:4D
4 nM	Other	–	18.7%	Other
		Ser_{162}/Tyr_{163}		
10 nM	Wild-type	...VSYGKV--KLVL...	0.9%	No variant
10 nM	Insertion	........L....	41.1%	-1:3I
10 nM	Insertion	........XQ...	3.5%	1:3I
10 nM	Deletion	......-..-...	2.1%	-7:6D
10 nM	Insertion	........X....	2.1%	-2:3I
10 nM	Insertion	.....AME....	1.7%	-3:1I,-2:5I
10 nM	Deletion	.........---.	1.5%	-4:9D
10 nM	Insertion	......-VV....	1.4%	-5:3I
10 nM	SNV	....N........	1.1%	SNV:-5
10 nM	Insertion	.......TXQ...	1.0%	1:6I
10 nM	Frameshift	.........AG.	11,6%	-1:1I
10 nM	Frameshift	......-..-AG.	11,3%	-6:5D
10 nM	Frameshift	..--------WS	3,6%	-16:16D
10 nM	Frameshift	....--------.	2,0%	-11:14D
10 nM	Other	–	15,2%	Other
		Ser_{162}/Tyr_{163}		
20 nM	Wild-type	...VSYGKV--KLVL...	1.6%	No variant
20 nM	Insertion	........L....	39.0%	-1:3I
20 nM	Insertion	........XQ...	9.5%	1:3I
20 nM	Deletion	.........---.	2.1%	-4:9D
20 nM	Insertion	........X....	1.9%	-2:3I
20 nM	Insertion	.......TL....	1.6%	-1:6I
20 nM	Insertion	.....AME....	1.5%	-3:1I,-2:5I
20 nM	Frameshift	..........AG.	9.3%	-1:1I
20 nM	Frameshift	.........-AG.	9.0%	-6:5D
20 nM	Frameshift	..----..--WS	4.2%	-16:16D
20 nM	Frameshift	.........-SWS	2.2%	-1:1D
20 nM	Frameshift	.........XAG.	1.8%	1:1I
20 nM	Frameshift	.........--WS	1.3%	-2:4D
20 nM	Frameshift	.........---T	1.0%	1:7D
20 nM	Frameshift	.........----	1.0%	-1:10D
20 nM	Other	–	13.0%	Other

Selection (Triptolide)	Type	Protein Sequence	Read Frequency	CrispRVariants Identifier
		Ser_{162}/Tyr_{163}		
2 nM	Wild-type	VSYGKV-KLVL	82,4%	No variant
2 nM	Insertion	.......L....	4,5%	-1:3I
2 nM	MNV	......D-Q...	1,6%	SNV:-3,-1
2 nM	Deletion	......----.	1,0%	-4:9D
2 nM	Frameshift	......---AG.	2,7%	-6:5D
2 nM	Frameshift	.......-.AG.	2,3%	-1:1I
2 nM	Other	–	5,5%	Other
		Ser_{162}/Tyr_{163}		
4 nM	Wild-type	VSYGKV--KLVL	14%	No variant
4 nM	Insertion	.......-L....	31,5%	-1:3I
4 nM	Deletion	.......-----.	6,2%	-4:9D
4 nM	MNV	......D--Q...	3,6%	SNV:-3,-1
4 nM	Insertion	.......-X....	2,9%	-2:3I
4 nM	Insertion	.......-TQ...	2,1%	1:3I
4 nM	Deletion	.......---M..	1,4%	1:3D
4 nM	Insertion	......AME....	1,1%	-3:1I,-2:5I
4 nM	Insertion	.......KTT...	1,1%	2:6I
4 nM	Frameshift	......----AG.	11,2%	-6:5D
4 nM	Frameshift	.......--.AG.	6,0%	-1:1I
4 nM	Frameshift	..--------WS	2,0%	-16:16D
4 nM	Frameshift	....--------.	1,7%	-11:14D
4 nM	Frameshift	.......--RAG.	1,3%	1:1I
4 nM	Frameshift	.......----WS	1,1%	-2:4D
4 nM	Frameshift	.......---SWS	1,0%	-1:1D
4 nM	Other	–	11,8%	Other
		Ser_{162}/Tyr_{163}		
10 nM	Wild-type	VSYGKV-----KLVL	0,6%	No Variant
10 nM	Insertion	......----L....	39,1%	-1:3I
10 nM	Insertion	......----TQ...	5,0%	1:3I
10 nM	Deletion	......--------.	2,7%	-4:9D
10 nM	Insertion	......----X....	2,6%	-2:3I
10 nM	Insertion	.....AME---....	1,9%	-3:1I,-2:5I
10 nM	Insertion	......----I....	1,6%	-3:3I
10 nM	Insertion	.....L----FQ...	1,5%	-4:3I
10 nM	Insertion	......KLWKSQ...	1,1%	1:15I
10 nM	Insertion	......--XXX....	1,0%	-1:9I
10 nM	Frameshift	.....-------AG.	8,5%	-6:5D
10 nM	Frameshift	......------.AG.	7,1%	-1:1I
10 nM	Frameshift	..-----------WS	4,0%	-16:16D
10 nM	Frameshift	......------SWS	3,9%	-1:1D
10 nM	Frameshift	....-----------.	3,1%	-11:14D
10 nM	Frameshift	......-----RAG.	1,7%	1:1I
10 nM	Frameshift	......-----TSWS	1,6%	-2:2I
10 nM	Stop	..---------*...	1,3%	-12:12D
10 nM	Frameshift	.....-----------	1,2%	-4:3D,2:10D
10 nM	Other	–	10,5%	Other
		Ser_{162}/Tyr_{163}		
20 nM	Wild-type	VSYGKV---KLVL	0,0%	No variant
20 nM	Insertion	.......--L....	37,6%	-1:3I
20 nM	Insertion	.......--I....	4,5%	-3:3I
20 nM	Deletion	......-----...	3,8%	-7:6D
20 nM	Insertion	.......--MR...	2,4%	1:2I,2:1I
20 nM	Insertion	.....A--GQ...	2,1%	-3:1I,-2:1I,1:1I
20 nM	Insertion	.......--TQ...	2,0%	1:3I
20 nM	Insertion	.......--X....	1,8%	-2:3I
20 nM	Insertion	........-KF....	1,8%	-3:6I
20 nM	Insertion	....IWKSL....	1,7%	-13:7I,-1:2I
20 nM	Deletion	.......------M	1,6%	1:9D
20 nM	Insertion	.......-XX....	1,3%	-1:6I
20 nM	Frameshift & other	–	39,3%	Other

Supplementary Figure 3: ERCC3 protein variants present in genome edited and triptolide resistant cells.
ERCC3 protein variants detected in HAP1 cells transfected with SpCas9 and sgRNA$_{ERCC3/K167}$. Transfected cells were treated with four different concentrations of triptolide and the experiment was repeated once. Protein variants were obtained by CrispRVariants analysis of next generation sequencing reads. Only variants detected above the 1% threshold are shown. Residues known to provide drug resistance upon mutation are highlighted in blue and the Cas9 cut site is shown with a red arrowhead and a vertical dotted line.

Supplementary Figure 4

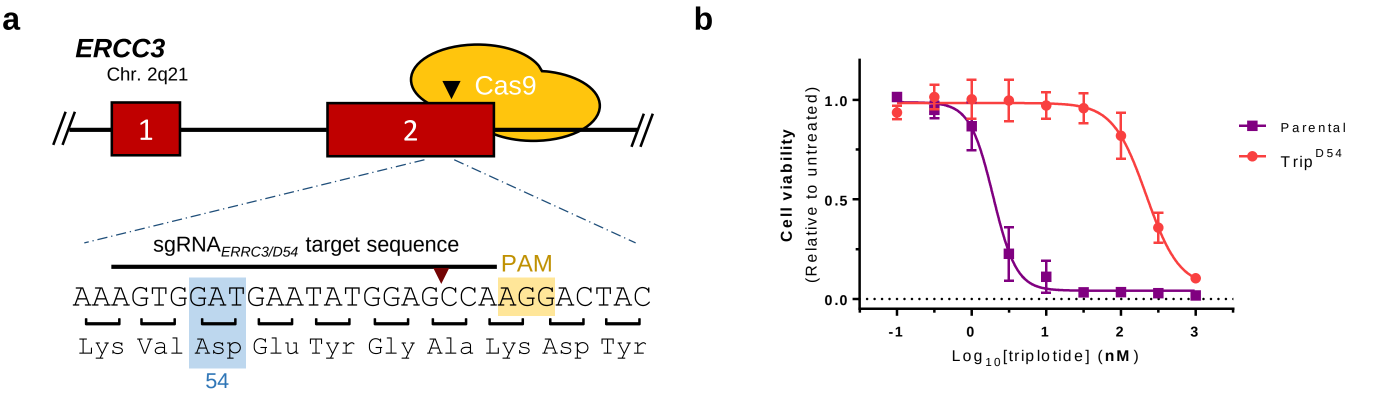

c

ERCC3^{D54}

Type	Protein Sequence (Asp$_{54}$)	Read Frequency	CrispRVariants Identifier
Wild-type	...KVDEYGAKDY...	0.5%	No variant
Deletion	...--.....	14.3%	-12:6D
Deletion	.---......	7.0%	-18:9D
Deletion	...---....	6.4%	-10:9D
Deletion	.-------..	4.6%	-21:21D
Deletion	...D-.....	2.6%	-9:3D
Deletion	----......	2.1%	-20:12D
Deletion	..-.......	1.6%	-13:3D
Deletion	..--......	1.6%	-14:6D
Deletion	...D---...	1.5%	-8:9D
Deletion	...------.	1.4%	-14:18D
Frameshifts and other	–	56.4%	Other

Supplementary Figure 4: generation of triptolide resistance mutations by CRISPR/Cas9-induced mutagenesis at the *ERCC3*^{D54} locus.

a. Schematic overview of the sgRNA targeting close to *ERCC3* aspartic acid$_{54}$, used for generation of triptolide resistance through Cas9 genome editing.

b. Cell viability assay showing the effect of triptolide on control and triptolide resistant, *ERCC3*^{D54} mutagenized HAP1 cells. Data points are normalized to untreated cells and represent means ± s.d. obtained from three experiments performed in triplicates. Cells were treated for 72h.

c. Amino acid sequences of Cas9/sgRNA$_{ERCC3/A58}$ transfected HAP1 cells treated with 10 nM triptolide as determined by CrispRVariants analysis of targeted amplicon sequencing reads. The Cas9 cut site is shown with a red arrowhead and a vertical dotted line, the aspartic acid$_{54}$ is highlighted with a blue column.

Supplementary Figure 5

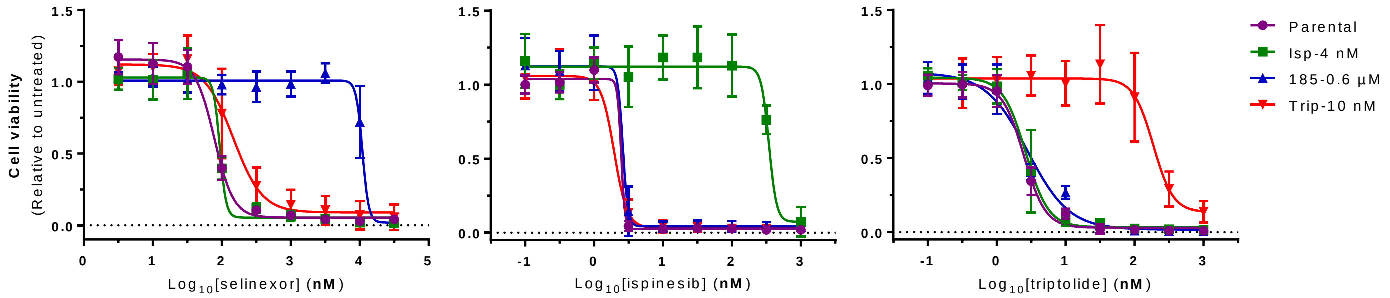

Supplementary Figure 5: assessment of cross-resistance between the different drug resistant and genome edited cells.
Cell viability assays showing the effect of selinexor, ispinesib or triptolide on wild-type (parental) and CRISPR/Cas9-mutagenized HAP1 cells. For each drug-resistant drug-target pair, cross-resistance against the other two compounds was assessed. Data points are normalized relative to untreated cells and represent averages ± s.d. obtained from three experiments performed in triplicate. Cells were treated for 72h.

Supplementary Figure 6

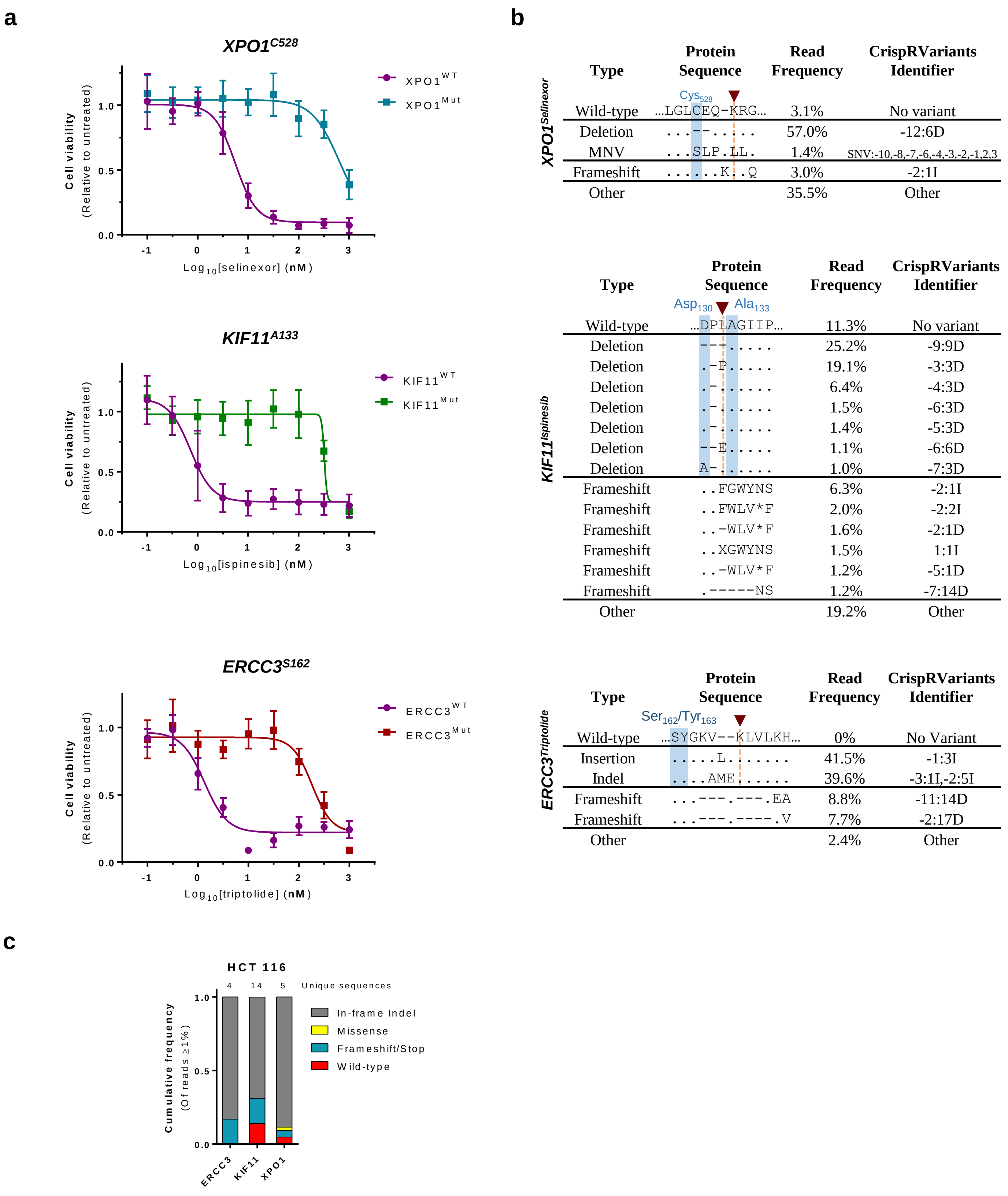

Supplementary Figure 6: generation of selinexor, ispinesib and triptolide resistant protein variants in HCT 116 cells by targeted CRISPR/Cas9-induced mutagenesis. Transfected cells were treated for 2 weeks with 1 µM selinexor (*XPO1*), 10 nM ispinesib (*KIF11*) or 20 nM triptolide (*ERCC3*) to obtain drug resistant cells. The same sgRNAs as shown in figure 1a were used for mutagenesis.

a. Cell viability after 72h of control and drug resistant, SpCas9 mutagenized HCT 116 cells in the presence of increasing concentrations of the respective drugs. Data points are normalized to untreated cells and represent means ± s.d. from three experiments performed in triplicate.

b. Amino acid sequences of the corresponding Cas9 mutagenized and drug resistant HCT 116 cells as determined by CrispRVariants analysis of targeted amplicon sequencing of the edited locus. Residues known to provide drug resistance upon mutation are highlighted with blue columns. The Cas9 cut site is highlighted by the vertical dotted line. Only variants detected with a read frequency ≥1% are shown.

c. Overview of the abundance of the mutation types detected in mutagenized and drug resistant HCT 116 cells. The relative abundance of each mutation type is shown and categorized per sample. Only one experiment per sample was performed. Results were obtained by targeted amplicon sequencing analysis with CrispRVariants of the genome edited locus (*ERCC3*: locus S162, *KIF11*: locus A133, *XPO1*: locus C528).

Supplementary Figure 7

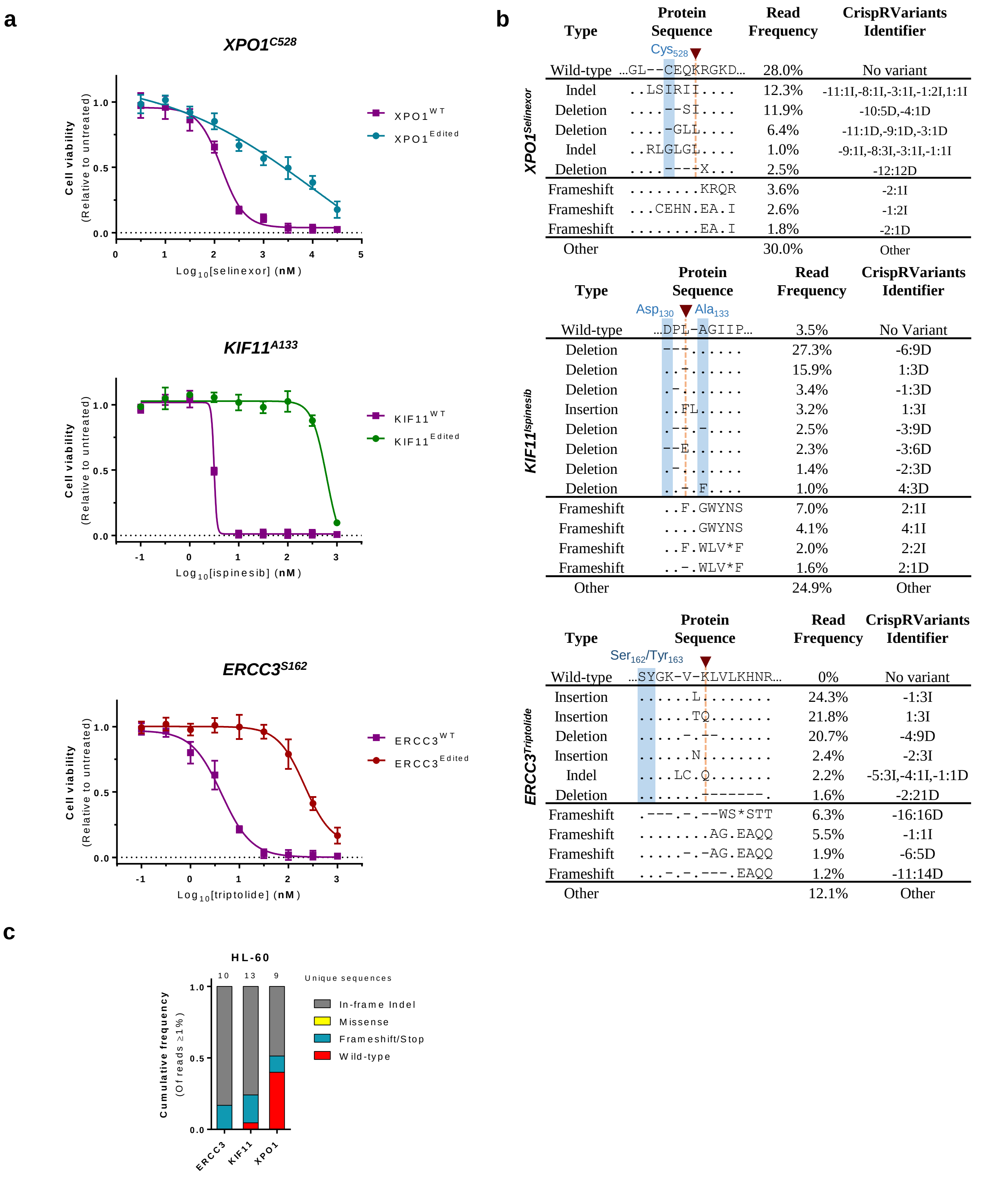

Supplementary Figure 7: generation of selinexor, ispinesib and triptolide resistant protein variants in HL-60 cells by CRISPR/Cas9-induced mutagenesis. Transfected cells were treated for 2 weeks with 1 µM selinexor (*XPO1*), 10 nM ispinesib (*KIF11*) or 20 nM triptolide (*ERCC3*) to obtain drug resistant cells. The same sgRNAs as shown in figure 1a were used for mutagenesis.

a. Cell viability after 72h of control and the different drug resistant and SpCas9 mutagenized HL-60 cells in the presence of the respective drugs. Data points are normalized to untreated cells and represent means ± s.d. obtained from two experiments performed in triplicate.

b. Amino acid sequences of the corresponding Cas9 mutagenized and drug resistant HL-60 cells as determined by CrispRVariants analysis of targeted amplicon sequencing of the edited locus. Residues known to provide drug resistance upon mutation are highlighted with blue columns. The Cas9 cut site is highlighted by the orange, vertical dotted line. Only variants with a read frequency ≥1% are shown.

c. Overview of the abundance of the different mutation types in the mutagenized HL-60 cells. The relative abundance of each type is shown and categorized per sample and only 1 experiment was performed for each sample. Results were obtained by targeted amplicon sequencing analysis with CrispRVariants of the genome edited locus in selected cells (*ERCC3*: locus S162, *KIF11*: locus A133, *XPO1*: locus C528).

Supplementary Figure 8

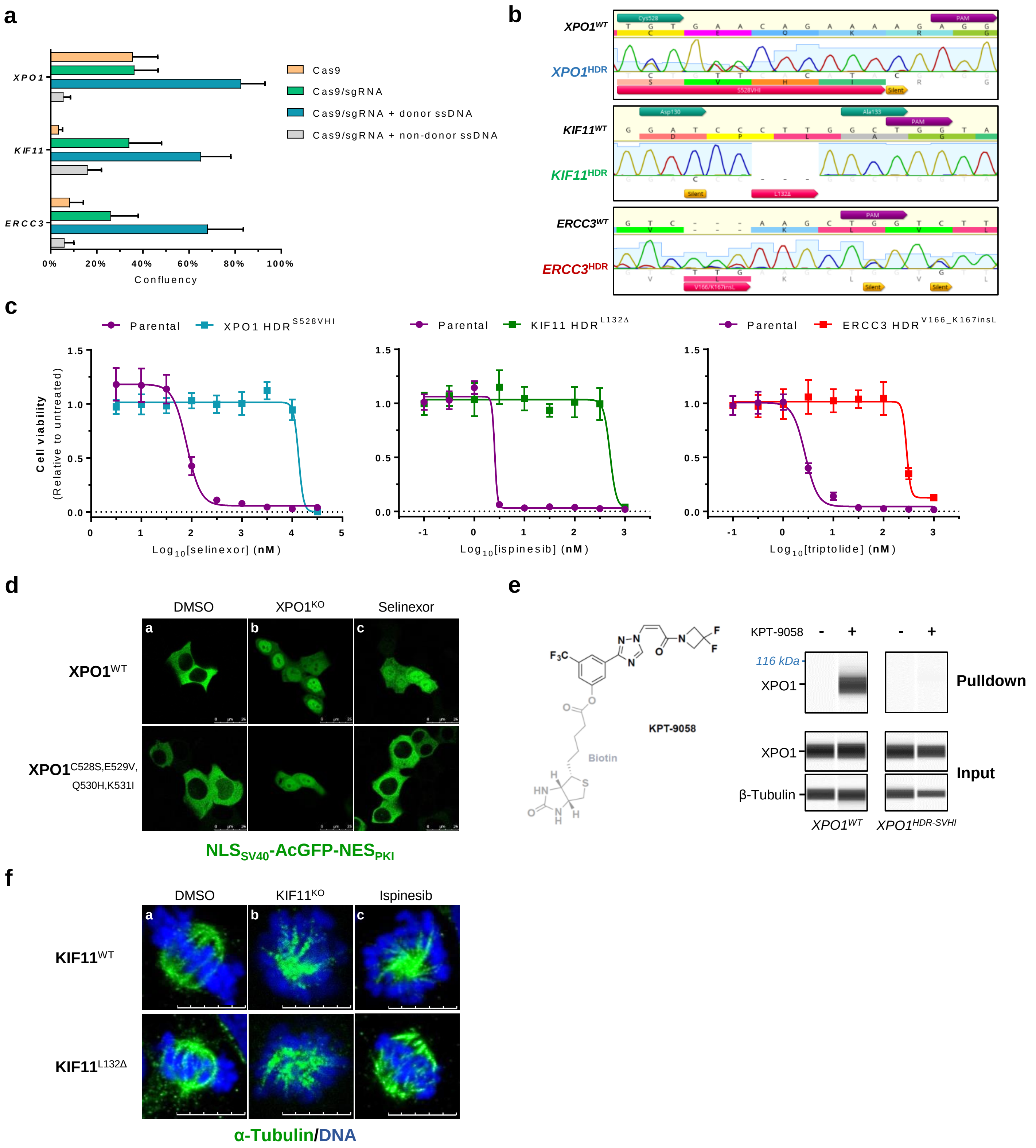

Supplementary Figure 8: validation of resistance mutations by CRISPR/Cas9-induced homology directed repair with ssDNA templates.

a. Viability of HAP1 cells transfected with either SpCas9 alone, Cas9 together with the resistant hotspot targeting sgRNA, Cas9 with the sgRNA and a ssDNA donor template, or Cas9 with the sgRNA and a non-donor ssDNA template after treatment for 5 days with selinexor (2 μM, *XPO1*), ispinesib (10 nM, *KIF11*) or triptolide (40 nM, *ERCC3*), respectively. Data represent the average confluency with standard deviation of a single well as visualized by 16 images taken of the same well by an IncuCyte ZOOM. Cells were treated 2 days after transfection.

b. Sequencing chromatograms of HDR-edited HAP1 cells after treatment. Silent mutations were incorporated to control for HDR.

c. Cell viability assays showing the effect of selinexor, ispinesib or triptolide on wild-type (parental) and respective HDR-edited HAP1 cells after 72h. Data points are normalized to untreated cells and represent averages ± s.d. obtained from two experiments performed in triplicate.

d. Confocal fluorescence imaging of parental and HDR-edited XPO1 HAP1 cells transfected with the NLS-AcGFP-NES XPO1 cargo (green). In addition, some cells were cotransfected with SpCas9 and a sgRNA pool targeting *XPO1* to induce knockout of XPO1 (panel b). Two days after transfection, cells were treated with DMSO (panel a) or 2 μM selinexor (panel c) for 3 hours and then imaged. Scale bars: 25 μm.

e. Pulldown of XPO1 from wild-type and mutant $XPO1^{HDR-S528VHI}$ HAP1 cells. Cells (2.5×10^6) were collected and lysed in RIPA buffer after treatment with DMSO or 2 μM KPT-9058 for 3 hours. KPT-9058 was extracted overnight at 4°C by streptavidin affinity chromatography (Dynabeads MyOne Streptavidin T1). The total lysate (input) and extracted fractions (pulldown) were then separated on a Simple Wes system. Indicated proteins were detected by chemiluminescent immunoblotting using primary and secondary-HRP conjugated antibodies.

f. Confocal immunofluorescence of mitotic spindle formation in parental or HDR-edited KIF11 HAP1 cells treated with DMSO (panel a) or 50 nM ispinesib (panel c) for 2 hours. Some cells were transfected with SpCas9 and a sgRNA pool targeting *KIF11* (panel b). Scale bars: 7.5 μm.

Supplementary Figure 9

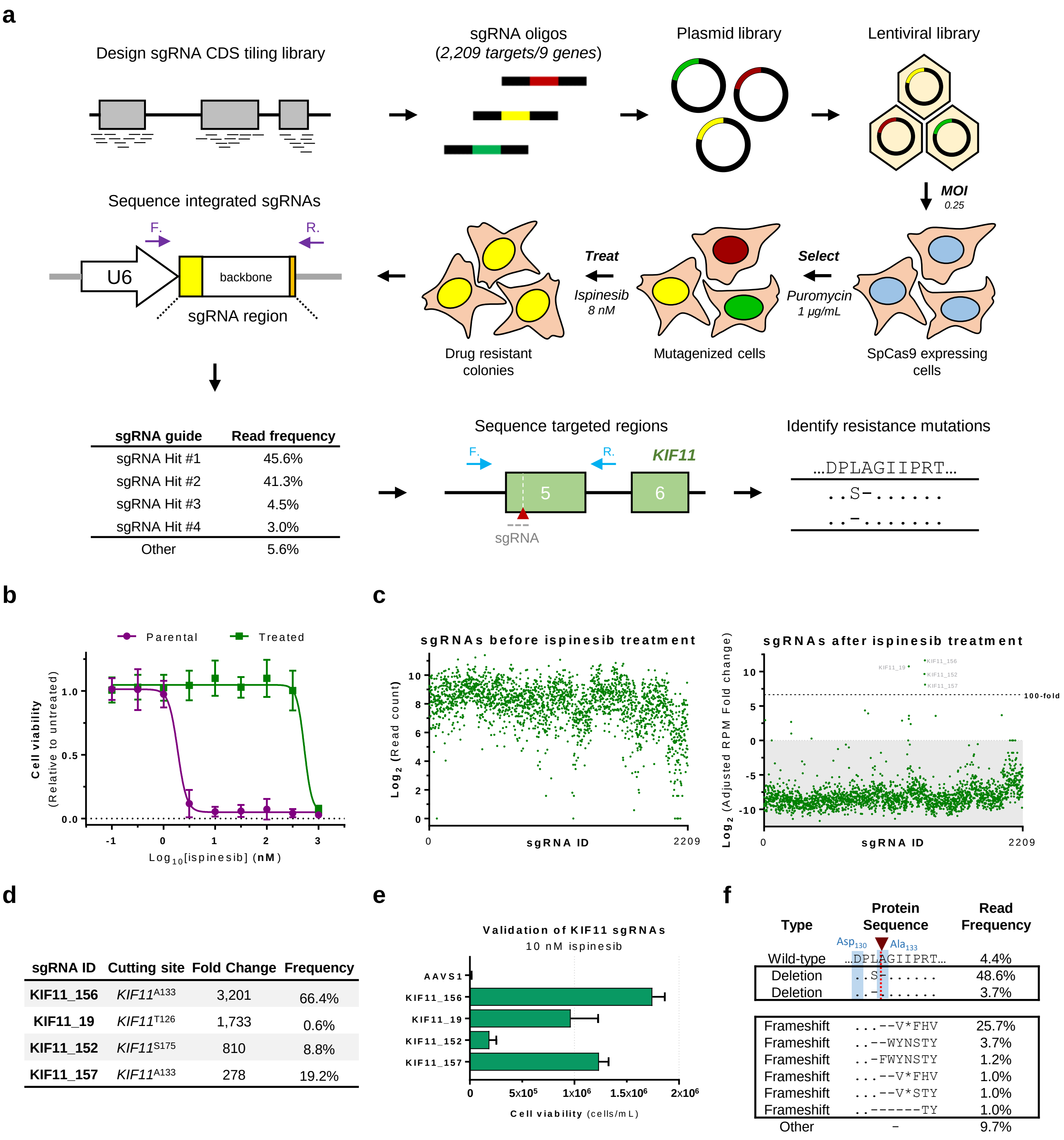

Supplementary Figure 9: proof of concept for the identification of a drug's target protein from a pool of 9 genes using the ispinesib-KIF11 drug-target interaction

a. Overview of the workflow for the CRISPR/Cas9-based chemical target identification screen used for ispinesib.

b. Cell viability after 72h of parental, Cas9⁺ and the polyclonal pool of drug resistant mutagenized HAP1 cells treated with different concentrations of ispinesib. Data points represent means ± standard deviation obtained from two experiments performed in triplicate.

c. Representation of the different sgRNAs in cells before (after puromycin selection) and after treatment with 8 nM ispinesib as determined by EdgeR analysis of next generation sequencing data. Each dot represents a different sgRNA and a value of 1 was added to the read count to facilitate log transformation. Fold change was determined by dividing the adjusted read count per million reads (RPM) of the sgRNAs enriched after ispinesib treatment by the adjusted read count per million reads of the sgRNAs detected after puromycin selection.

d. Overview of sgRNA hits with a fold change >100 identified in surviving cells 14 days after ispinesib treatment. All sgRNAs targeted *KIF11.*

e. Enriched sgRNAs were cloned individually and transfected into parental HAP1 cells stably expressing SpCas9 to validate the results. Three days after transfection, cells were treated with 10 nM ispinesib for 5 days and surviving cells were counted using trypan blue exclusion. An sgRNA targeting the AAVS1 safe harbor locus on chromosome 19q13.42 was used as negative control. Values represent means ± s.d. obtained from two independent experiments.

f. Amino acid variants detected in the KIF11 ala133 locus of the resistant pool of cells. The asp130 and the ala133 residue are highlighted and the sgRNA cut site (KIF11_156/157) is highlighted with a red arrowhead. Alterations were uncovered by CrispRVariants analysis of next generation sequencing data. Only variants with a frequency above 1% are shown.

Supplementary Figure 10

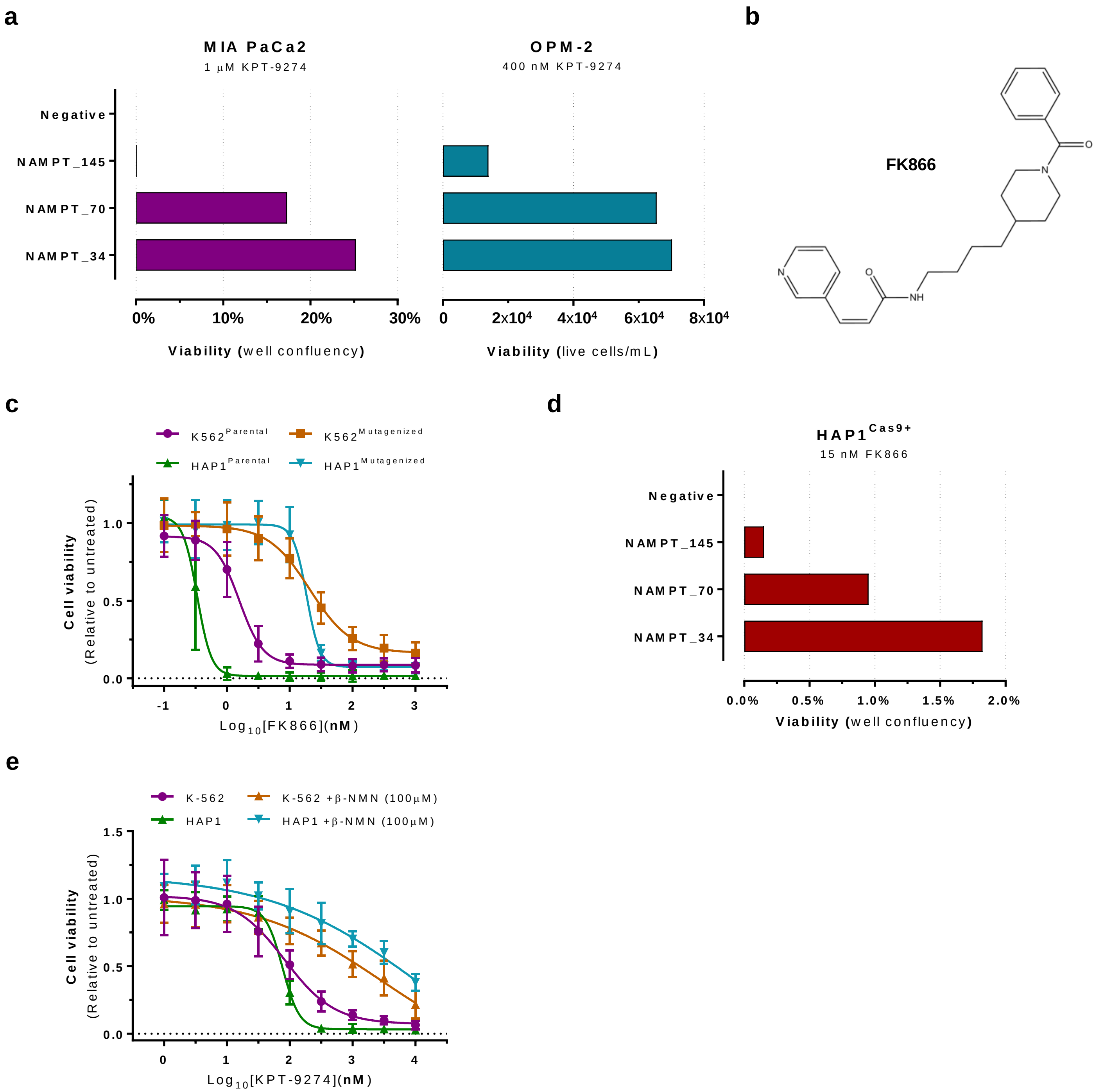

Supplementary Figure 10: the enriched NAMPT sgRNAs confer resistance to multiple myeloma cells and KPT-9274 resistant cells are cross-resistant to the classical NAMPT inhibitor FK866.

a. Selection of KPT-9274 resistance in MIA PaCa2 and OPM-2 cells using highly enriched NAMPT sgRNAs identified in the KPT-9274 screen. Cells were cotransfected with the indicated NAMPT sgRNAs and SpCas9. Two to three days after transfection, cells were treated for 8 days (MIA PaCa2) or 22 days (OPM-2) with the indicated concentration of KPT-9274. For MIA PaCa2 cells, confluency was measured using an IncuCyte Zoom. For OPM-2 cells, the amount of surviving cells was measured by trypan blue exclusion cell counting.

b. Chemical structure of FK866.

c. The cell populations that survived the mutagenesis screen with KPT-9274 are cross-resistant to the NAMPT inhibitor FK866. Cell viability assays show the effect of FK866 on parental HAP1 cells stably expressing SpCas9 and the polyclonal mutagenized KPT-9274 resistant cells obtained from the CRISPR/Cas scanning screen. Data points were obtained 72h after addition of the compound, were normalized relative to untreated cells and represent averages ± s.d. obtained from two (HAP1) or five (K-562) experiments performed in triplicate.

d. NAMPT sgRNAs enriched in the KPT-9274 mutagenesis screen confer FK866 resistance to HAP1 cells. Parental HAP1 cells stably expressing SpCas9 were transfected with the indicated sgRNAs and treated with 15 nM FK866 for a period of 8 days. Cell confluency was then measured using an IncuCyte Zoom.

e. Cell viability assay showing the protective effect of β-nicotinamide mononucleotide (β-NMN) on parental HAP1 cells stably expressing SpCas9 in the presence of increasing concentrations of KPT-9274. Data points were obtained after 72h, normalized to untreated cells and represent averages ± s.d. obtained from two (HAP1) or four (K-562) experiments performed in triplicate.

Supplementary Figure 11

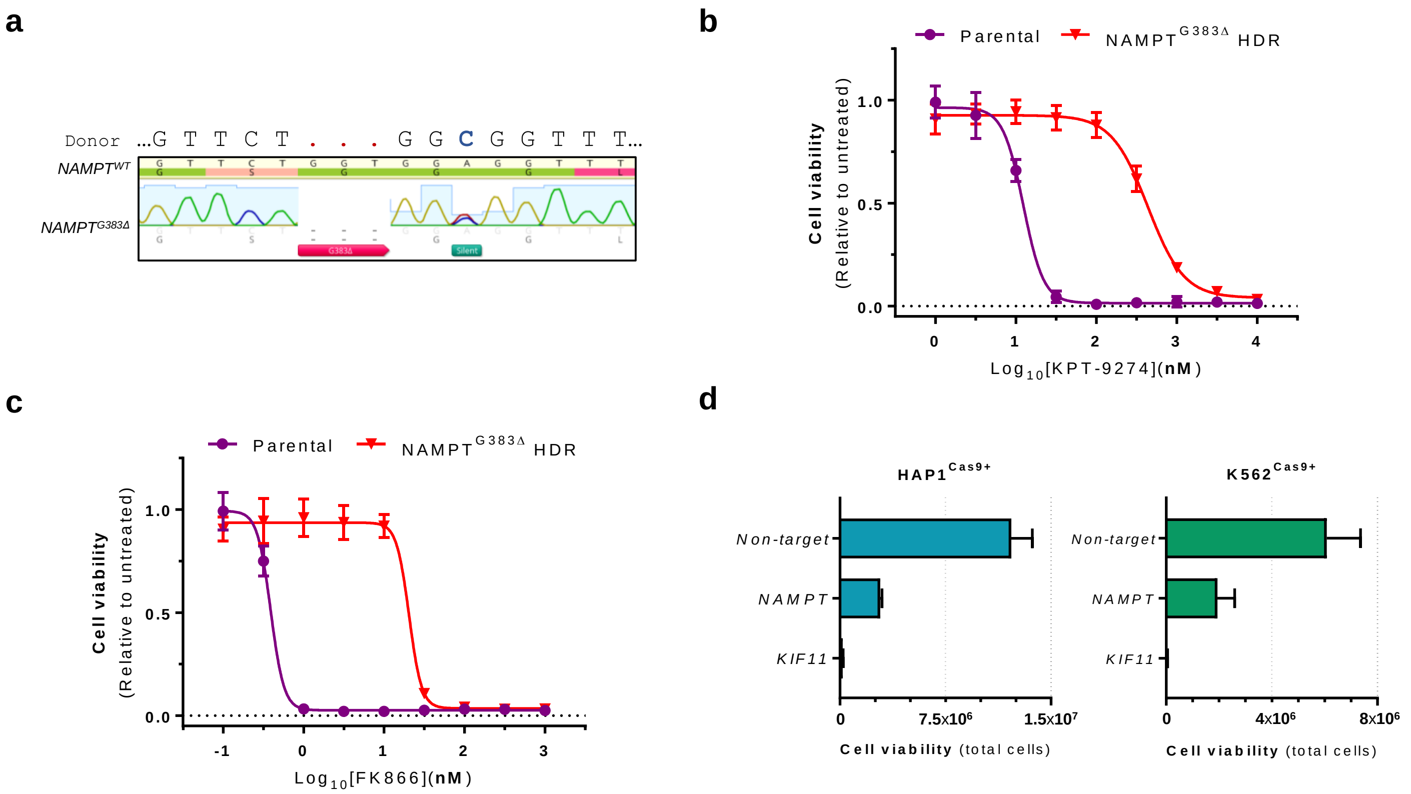

Supplementary Figure 11: validation of KPT-9274 resistance mutations in NAMPT by CRISPR/Cas9-induced HDR.

a. Sequencing chromatogram of *NAMPT* in HDR-edited HAP1 cells stably expressing SpCas9. Cells were transfected with a *NAMPT* targeting sgRNA and a HDR donor template containing the G383del mutation together with a silent mutation not identified in our screen to control for HDR. Two days after transfection, cells were treated with 300 nM KPT-9274 for 1-2 weeks.

b. Cell viability assay showing the effect of KPT-9274 on wild-type, Cas9+ (parental) and polyclonal G383del HDR-edited HAP1 cells after 72h. Data points are normalized to untreated cells and represent averages ± s.d. obtained from three experiments performed in duplicate.

c. Cell viability assay showing the effect of FK866 on wild-type, Cas9+ (parental) and polyclonal G383del HDR-edited HAP1 cells after 72h. Data points are normalized to untreated cells and represent averages ± s.d. obtained from three experiments performed in triplicate.

d. Knockout of NAMPT using CRISPR/Cas9-mediated genome editing shows that NAMPT is important for cell survival. HAP1 or K-562 cells stably expressing SpCas9 were transfected with sgRNA plasmid pools targeting the indicated gene. The following day, cells were treated for 24 hours with puromycin to select for transfected cells. Four days after selection, surviving cells were counted using trypan blue exclusion. *KIF11* is an essential gene and served as positive control, while non-targeting sgRNAs were used as negative control. Values represent means ± s.d. obtained from two independent experiments.

Supplementary Figure 12

a

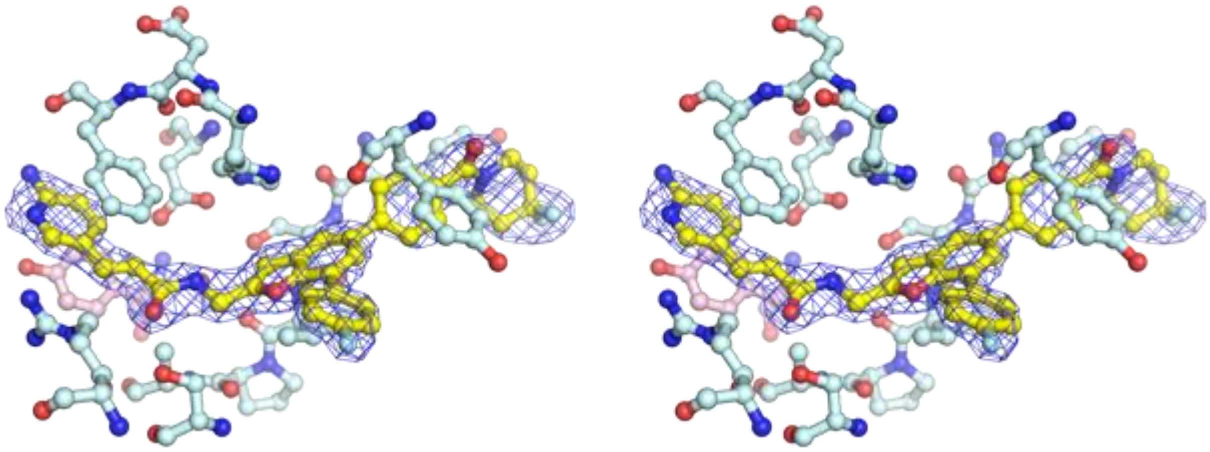

b

	Human NAMPT + KPT-9274
**Data collection**	
Space group	$P2_1$
Cell dimensions	
a, b, c (Å)	62.14, 109.17, 84.97
α, β, γ (°)	90.00, 96.63, 90.00
Resolution (Å)	50.00 – 2.05 (2.12 – 2.05)
R_{merge}	0.139 (0.557)
I / σI	9.0 (2.1)
Completeness (%)	100.0 (99.8)
Redundancy	3.8 (3.5)
**Refinement**	
Resolution (Å)	42.20 – 2.05
No. reflections	67,850
R_{work} / R_{free}	0.235 / 0.283
No. atoms	
Protein	7,478
Ligand/ion	184
Water	809
B-factors	
Protein	25.04
Ligand/ion	39.27
Water	31.15
Ramachandran Plot	
Favored (%)	96.75
Allowed (%)	3.25
R.m.s. deviations	
Bond lengths (Å)	0.006
Bond angles (°)	0.66

Supplementary Figure 12: crystallization of KPT-9274 with NAMPT dimer.

a. The electron density map of the ligand (yellow) in the NAMPT binding site at 1σ shows a perfect match between the ligand and the observed density.

b. Data collection and refinement statistics (molecular replacement).

Supplementary Figure 13

a

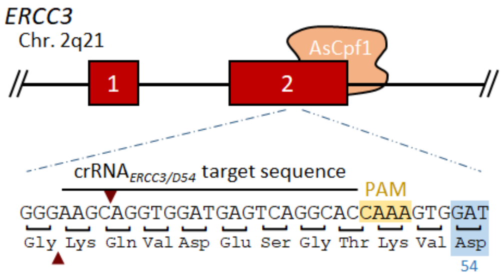

b

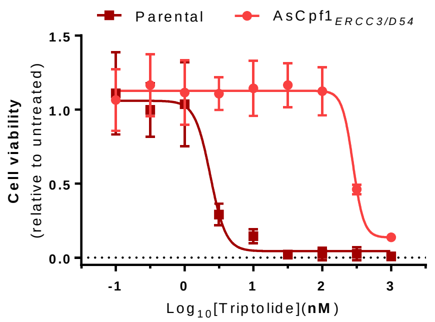

c

Type	Amino Acid Sequence	Read Frequency	CrispRVariants Identifier
Wild-type	...AAGKQVDES...	0%	no variant
Deletion	.....-...	12.8%	2:3D
Deletion	....-L...	8.6%	1:3D
Deletion	....-....	7.8%	-3:3D
Deletion	....--H..	4.6%	1:6D
Deletion	..----...	3.7%	-8:12D
Frameshift	.....-MSQ	6.6%	2:4D
Frameshift	....--MSQ	4.0%	-2:7D
Other	-	37.3%	Other

Supplementary Figure 13: generation of triptolide resistance by AsCpf1-mediated genome editing near the ERCC3^{D54} locus.

a. Schematic overview of AsCpf1-mediated mutagenesis near the ERCC3^{D54} locus. Asp54, known to provide resistance upon mutation is highlighted in blue. The crRNA cutting site is denoted by red arrows.

b. Cell viability after 72h of wild-type parental and polyclonal mutagenized HAP1 cells treated with different concentrations of triptolide. Signals are plotted relative to the untreated control and data points represent means ± s.d. obtained from three experiments performed in triplicate.

c. Amino acids variants identified in the triptolide resistant HAP1 cells mutagenized at the ERCC^{54} locus. Only reads with a read frequency above 3% are shown. Variants were obtained by CrispRVariants analysis of next-generation sequencing reads. Amino acids Ala41 to Ser49 are shown and no mutations were detected at Asp54.

Supplementary Figure 14

a

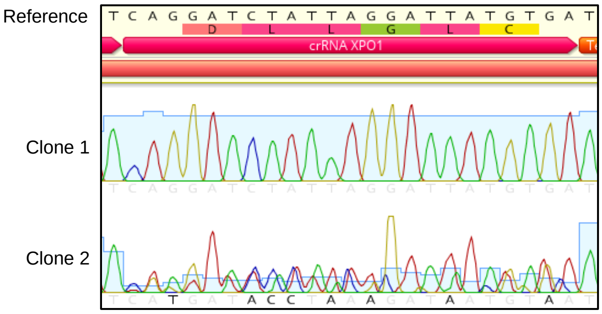

b

crRNA sequence	Cpf1 cutting site	Frequency
CAGGATCTATTAGGATTATGTGA	XPO1^{C528}	37,8%
CAGGATCTATTAGGATTATGTGA<br>AGTAAAACCAACACAGAACAGAC	XPO1^{C528}<br>RSP3a^{F138}	20,0%
CAGGATCTATTAGGATTATGTGA<br>CCAGCA**T**TCCTTGCTATTCCACC	XPO1^{C528}<br>XPO1^{P867}	20,0%
CAGGATCTATTAGGATTATGTGA<br>C**T**TACCTTCGCACTGGTTCCTTG	XPO1^{C528}<br>XPO1^{R96}	17,8%
CAGGATCTATTAGGATTATGTGA<br>TTCTTTATTTTCAGTAATCTATG	XPO1^{C528}<br>XPO1^{M1}	2,2%
CAGGATCTATTAGGATTATGTGA<br>CTTTCTTAGAGATCTTGCTGCCC	XPO1^{C528}<br>ABL1^{A385}	2,2%

Supplementary Figure 14: crRNA sequencing results of single-cell derived selinexor resistant colonies obtained from the pool of resistant cells.

a. Sequencing chromatogram of sgRNAs present in single-cell derived selinexor resistant HAP1 colonies as obtained by Sanger sequencing. Each chromatogram denotes a different clone. The reference sequence above contains the *XPO1*^{C528} codon targeting crRNA sequence. The first clone contains a single crRNA targeting codon *XPO1*^{C528}. The second clone contains 2 crRNAs, one targeting codon *XPO1*^{C528} and one targeting codon *RSP3a*^{F138} (AGTAAAACCAACACAGAACAGAC).

b. All single-cell derived clones contained the *XPO1*^{C528} targeting crRNA and some clones were transduced with 2 crRNAs. Some mismatches in the AsCpf1 guide sequences were detected and are underlined.
