# Supplementary material for: Target identification of small molecules using large-scale CRISPR-Cas mutagenesis scanning of essential genes
Source: Nat Commun. 2018 Feb 5;9:502. doi: 10.1038/s41467-017-02349-8 (PMC5799254; doi:10.1038/s41467-017-02349-8)
Supplement: Supplementary file 2 — Description of Additional Supplementary Files [file 41467_2017_2349_MOESM2_ESM.pdf]

Description of Additional Supplementary Files

File Name: Supplementary Data 1

Description: list of the 2,209 sgRNAs present in the SpCas9 tiling library covering 9 genes.

File Name: Supplementary Data 2

Description: list of read counts of the sgRNAs present in HAP1Cas9+ cells transduced with the 9 gene SpCas9 tiling library before and after treatment with 8 nM Ispinesib.

File Name: Supplementary Data 3

Description: list of the 40,517 sgRNAs present in the two SpCas9 FDA targets tiling sublibraries A and B, together covering 115 genes.

File Name: Supplementary Data 4

Description: list of the 24,435 sgRNAs present in the two SpCas9 Non-FDA targets tiling sublibraries C and D, together covering 75 genes.

File Name: Supplementary Data 5

Description: list of read counts of the sgRNAs present in HAP1Cas9+ cells transduced with the FDA tiling sublibrary B for SpCas9 before and after treatment with 30 nM Bortezomib.

File Name: Supplementary Data 6

Description: list of read counts of the sgRNAs present in HAP1Cas9+ and K-562Cas9+ cells transduced with the non-FDA tiling sublibraries C and D before and after treatment with 300 (HAP1) or 500 (K562) nM KPT-9274.

File Name: Supplementary Data 7

Description: uncovered NAMPT mutations in transduced K-562 and HAP1 cells after treatment with KPT-9274.

File Name: Supplementary Data 8

Description: list of the 1,100 crRNAs present in the AsCpf1 tiling library covering 10 genes

File Name: Supplementary Data 9

Description: list of read counts of the sgRNAs present in HAP1Cas9+ cells transduced with the AsCpf1 tiling library before and after treatment with 2 µM selinexor (KPT-330).

File Name: Supplementary Data 10

Description: overview of the primers, HDR templates and crRNA/sgRNA sequences used throughout the manuscript

File Name: Supplementary Software

Description: R Scripts for sequence data analysis
